# Systematic review of reviews of intervention components associated with increased effectiveness in dietary and physical activity interventions

**DOI:** 10.1186/1471-2458-11-119

**Published:** 2011-02-18

**Authors:** Colin J Greaves, Kate E Sheppard, Charles Abraham, Wendy Hardeman, Michael Roden, Philip H Evans, Peter Schwarz

**Affiliations:** 1 University of Exeter, Peninsula Medical School, Smeall Building, St Luke's Campus, Magdalen Road, Exeter EX1 2LU, UK; 2 University of Sussex, School of Psychology, Pevensey Building, Falmer BN1 9QG, UK; 3 University of Cambridge, General Practice and Primary Care Research Unit, 16 Colwyn Close, Cambridge CB4 3NU, UK; 4 Heinrich-Heine University, Institute for Clinical Diabetology, German Diabetes Centre and Department of Metabolic Diseases, Auf'm Hennekamp 65, 40225 Düsseldorf, Germany; 5 University of Plymouth, Peninsula Medical School, Smeall Building, St Luke's Campus, Magdalen Road, Exeter EX1 2LU, UK; 6 Technical University of Dresden (Carl Gustav Carus Medical Faculty), Medizinische Klinik III, Fetscherstraße 74, Dresden D-01307, Germany; 7 International collaboration group, led from Technical University of Dresden, c/o Prof Peter Schwarz, Medizinische Klinik III, Fetscherstraße 74, Dresden D-01307, Germany

## Abstract

**Background:**

To develop more efficient programmes for promoting dietary and/or physical activity change (in order to prevent type 2 diabetes) it is critical to ensure that the intervention components and characteristics most strongly associated with effectiveness are included. The aim of this systematic review of reviews was to identify intervention components that are associated with increased change in diet and/or physical activity in individuals at risk of type 2 diabetes.

**Methods:**

MEDLINE, EMBASE, CINAHL, PsycInfo, and the Cochrane Library were searched for systematic reviews of interventions targeting diet and/or physical activity in adults at risk of developing type 2 diabetes from 1998 to 2008. Two reviewers independently selected reviews and rated methodological quality. Individual analyses from reviews relating effectiveness to intervention components were extracted, graded for evidence quality and summarised.

**Results:**

Of 3856 identified articles, 30 met the inclusion criteria and 129 analyses related intervention components to effectiveness. These included causal analyses (based on randomisation of participants to different intervention conditions) and associative analyses (e.g. meta-regression). Overall, interventions produced clinically meaningful weight loss (3-5 kg at 12 months; 2-3 kg at 36 months) and increased physical activity (30-60 mins/week of moderate activity at 12-18 months). Based on causal analyses, intervention effectiveness was increased by engaging social support, targeting both diet and physical activity, and using well-defined/established behaviour change techniques. Increased effectiveness was also *associated with *increased contact frequency and using a specific cluster of "self-regulatory" behaviour change techniques (e.g. goal-setting, self-monitoring). No clear relationships were found between effectiveness and intervention setting, delivery mode, study population or delivery provider. Evidence on long-term effectiveness suggested the need for greater consideration of behaviour maintenance strategies.

**Conclusions:**

This comprehensive review of reviews identifies specific components which are associated with increased effectiveness in interventions to promote change in diet and/or physical activity. To maximise the efficiency of programmes for diabetes prevention, practitioners and commissioning organisations should consider including these components.

## Background

The development of type 2 diabetes is strongly associated with being overweight, obese or physically inactive[1,2]. Large randomised controlled trials (RCTs) have shown that relatively modest changes in lifestyle (increasing fibre (≥15 g/1000 kcal), reducing total fat (< 30% of energy consumed) and saturated fat (< 10% of energy consumed), engaging in moderate physical activity (≥30 mins/day), weight reduction (5%)) can reduce the risk of progression to type 2 diabetes in adults with impaired glucose regulation (also known as pre-diabetes) by around 50%[3-7]. In one study, achieving four or more of the above targets led to zero incidence of type 2 diabetes up to seven years later[8]. Consequently, promoting changes in physical activity and dietary intake is now recommended in national and international guidelines as a first line therapy for preventing type 2 diabetes[9-12].

A number of diabetes prevention programmes have been developed internationally (e.g. in Finland,[13] Germany,[14,15] the US,[16,17] Australia[18] and China[19]). However, national diabetes prevention strategies are still lacking in many countries. The cost-effectiveness of lifestyle intervention approaches for diabetes prevention is already well established and is favourable in comparison to pharmacological approaches[20-22]. However, most interventions used to date in a research setting are considered to be too intensive for widespread implementation in health services[23]. For example, the US Diabetes Prevention Programme[4] involved 16 individual counselling sessions plus individual coaching and a maintenance programme with further individual and group sessions. A major challenge for healthcare providers therefore is how to achieve the lifestyle changes needed to prevent type 2 diabetes (and its associated cardiovascular risk) without overstretching existing budgets and available resources[24,25].

In translating the research evidence into practical programmes it is critical to ensure that the intervention components (i.e. behaviour change techniques and strategies) and characteristics (e.g. setting, delivery mode, intervention provider) most strongly associated with effectiveness are included.

We therefore aimed to systematically review existing systematic reviews to summarise the evidence relating the content of interventions for promoting dietary and/or physical activity change to their effectiveness in producing weight and behaviour change. The review focused on evidence relating to individuals at risk of type 2 diabetes due to lifestyle (e.g. inactivity) or clinical risk factors (e.g. overweight, elevated blood pressure).

## Methods

### Data Sources and Search Strategy

One author (KS) searched MEDLINE, EMBASE, CINAHL, PsycInfo, and the Cochrane Library for systematic reviews in the English language, published between January 1998 and May 2008 (the search terms were reviewed by several authors (CG, CA, WH) and are provided in Additional file 1 Table S1). Reference lists of selected reviews and relevant clinical guidelines were also searched and experts in the area were contacted in order to identify unpublished reviews.

### Review selection

Two reviewers (KS, CG) independently examined titles and abstracts. Relevant review articles were obtained in full, and assessed against the inclusion and study quality criteria described below. Inter-reviewer agreement on inclusion was assessed using kappa statistics and any disagreements were resolved through discussion.

#### Inclusion criteria

1) Type of study: Systematic reviews and meta-analyses including RCTs, observational studies, case-controlled or other quasi-experimental studies. Comparison groups could include usual care, no intervention or other interventions. 2) Type of intervention: Interventions promoting physical activity and/or dietary change at the individual-level (i.e. interventions delivered to individuals either singly or in group sessions, but not whole-community or whole-population level interventions such as media campaigns or changes in the local environment). 3) Study populations: Adults (18 years and over) at risk of developing type 2 diabetes, selected because they were obese, overweight, sedentary, had hypertension, impaired fasting glucose, impaired glucose tolerance, hyperlipidaemia, metabolic syndrome, polycystic ovarian syndrome, gestational diabetes, a family history of type 2 diabetes or cardiovascular disease, or had been identified as having a high cardiovascular disease risk score (e.g. using a validated risk score such as Q-RISK or Framingham).

#### Exclusion criteria

1) Reviews not meeting pre-defined criteria for methodological quality (Additional file 1 Table S2). 2) Reviews which focused on people with existing diabetes, cardiovascular disease, or solely on healthy adults, or which were confined to groups with significant co-morbidities (e.g. arthritis, mental health).

#### Outcomes

We selected reviews where the primary outcome measure was weight, weight loss (kg or Body Mass Index (BMI), proportions of people achieving a target weight loss), changes in physical activity (e.g. frequency, met-hrs per week) or dietary behaviour. Behaviours could be measured objectively (e.g. with accelerometers) or by self-report (e.g. dietary intake questionnaires). Cardio-respiratory fitness was considered as a proxy for change in physical activity. As self-report increases the risk of measurement bias,[26,27] we have highlighted findings based on self-report in the data tables (Additional file 2 Tables S7-S14). We also examined papers for other outcomes which might be of interest in relation to change in weight, diet, or physical activity behaviour or in relation to the progression to type 2 diabetes.

### Study quality assessment

Review quality was rated independently by two authors (KS, CG) for a sub-sample (35 out of 107) of the articles identified as potentially relevant, using the Overview Quality Assessment Questionnaire (OQAQ;[28] Additional file 1 Table S2). Thereafter, review quality was rated by one researcher (KS) and verified by another (CG). Reviews were included if their OQAQ score was 14 or more (possible range 0-18) and if they scored at least one point for either of the two OQAQ criteria about assessing quality/taking quality into account in analyses (this was intended to maximise the likely quality of evidence underlying the review-level analyses). A percentage score was calculated for inter-rater agreement (defined as ≤1 point of variation on OQAQ scores) and any disagreements were resolved by discussion.

### Data extraction

We extracted data on the effectiveness of interventions and on the relationship of effectiveness to seven pre-defined intervention components. These were: Theoretical basis (i.e. we extracted analyses relating effectiveness to the use of any stated theory of behaviour or behaviour change); Behaviour change techniques used (e.g. the use of specific techniques such as goal-setting, problem-solving or the planned use of some clearly defined set of behaviour change techniques: See Table 1 for examples); Mode of delivery (e.g. group-based, individual, self-delivery, mixed-mode); Intervention provider (e.g. general practitioner, counsellor); Intensity (e.g. number of sessions, total contact time); Characteristics of the target population (e.g. age, ethnicity, risk state); and Setting (e.g. primary care, workplace). Data were extracted against a data extraction template by one author (KS) and checked by another (CG) with reference to the full text of the article. Extracted data also included inclusion and exclusion criteria, reported analyses and analysis type.

**Table 1:** Definitions of 'established behaviour change techniques'

Source	Basis for categorisation
Avenell et al. 2004 [31]	Definitions of behaviour therapy varied by study but include self-monitoring, stimulus control, problem solving, relapse prevention management, cognitive restructuring, self-assertion, social support, goal setting, self-reinforcement.

McTigue et al. 2003 [46]	Behavioural interventions are strategies to help patients acquire the skills, motivations, and support to change diet and exercise patterns. These include barrier identification, problem solving, self-monitoring, social support, goal-setting, developing action plans, relapse prevention, stimulus control and cognitive restructuring.

Shaw et al. 2005 [54]	Behavioural therapy aims to provide the individual with coping skills to handle various cues to overeat and to manage lapses in diet and physical activity when they occur and to provide motivation essential to maintain adherence to a healthier lifestyle once the initial enthusiasm for the programme has waned. Therapeutic techniques in studies relating to the benefit of using "established behaviour change techniques" include stimulus control, self-control and therapist-controlled contingencies, self-monitoring, problem solving, goal setting, behaviour modification, reinforcement.

NICE Obesity guidance [67]	This guidance document comprises a summary (and expansion) of reviews by Shaw et al.[54], McTigue et al.[46], Avenell et al.[31] and Smith et al.[71]. Definitions vary by analysis but typically include cue avoidance, self-monitoring, stimulus control, social support, planning problem solving, cognitive restructuring, modifying thoughts, relapse prevention, reinforcement of change, coping strategies, coping imagery, goal setting, social assertion, reinforcement techniques for enhancing motivation.

### Grading of evidence

An evidence grade was given to each reported analysis, based on the Scottish Intercollegiate Guidelines Network (SIGN) evidence grading system[29]. This system grades the risk of bias associated with a particular piece of evidence on a hierarchy from meta-analysis and RCT evidence (grade 1) down to expert opinion (grade 4), with additional indicators (++, + or -) to indicate methodological quality. The SIGN system was modified, as our review aimed to identify the relative effectiveness of intervention components, rather than effectiveness *per se *(see Additional file 1 Table S3 for full details). Although the SIGN evidence grading uses an alpha-numeric system (1++, 1+, 1-, 2++, 2+, 2-), for ease of reading we have converted this to a text-based format. For each analysis the quality of the evidence (the degree of confidence that the risk of bias is low) is described as either "high (++), medium (+) or low (-)". Each analysis is also categorised as being either "causal" evidence (SIGN grade 1; evidence from meta-analyses or summaries of RCTs where the component or characteristic of interest was experimentally manipulated) or "associative" evidence (SIGN grade 2; evidence from correlational or observational analyses). We also applied a category of "very low quality" for analyses with very low apparent power (total N < 100). The reporting that follows excludes this very low quality evidence, although it is included in the supplementary data tables for completeness.

### Analysis

No statistical analyses or meta-analyses were conducted. Instead, the existing analyses reported in the articles reviewed were extracted and reported in a systematic format (Additional file 2 Tables S7 to S14). Each analysis was graded using the adapted SIGN criteria as described above and a narrative synthesis is presented below, indicating both the quality of the evidence (low, medium, high) and whether it is causal or associative in nature.

In accordance with reporting guidelines for systematic reviews, a PRISMA (Preferred Reporting Items for Systematic Reviews and Meta-Analyses) checklist is available for this review (Additional file 3).

## Results

**Figure 1:**
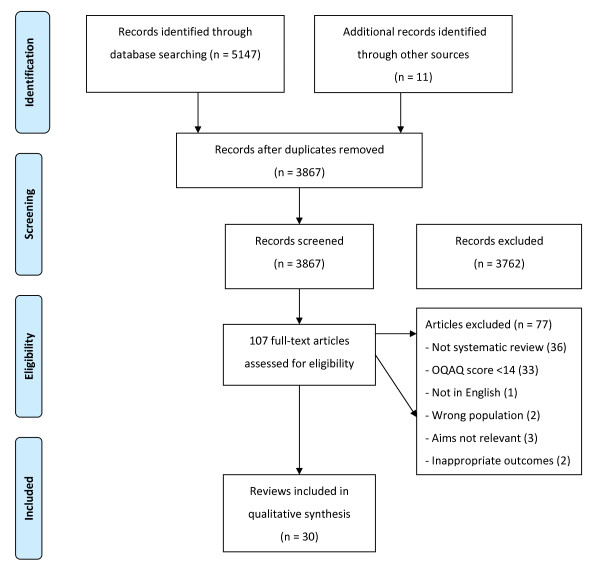
**Flow diagram of study selection**.

Searches identified 3856 potentially relevant articles. Following review of titles and abstracts, 96 articles were retrieved and quality-assessed. An additional 11 articles were identified through reference lists and grey literature. Of these 107 articles, 30 met both the selection and quality criteria (Figure 1) and these are identified by an asterisk in the reference list[30-59]. The inter-rater reliability (Kappa) for applying review selection criteria was 0.71 (95%CI: 0.61 to 0.80), and the proportion for inter-reviewer agreement on review quality was 0.70 (95%CI: 0.55 to 0.85).

### Review characteristics

The characteristics of the included and excluded reviews are summarised in Additional file 1 Tables S4 and S5. Ten reviews examined physical activity interventions, three examined dietary interventions and seventeen examined both. Reviews included data from a range of populations (e.g. sedentary, overweight, obese, impaired glucose tolerance) and delivery settings (e.g. home based, leisure centre based, primary care, workplace) and used a variety of descriptive, meta-analytic and meta-regression analyses to investigate the association of intervention components with effectiveness. We identified 129 analyses of relationships between intervention components and effectiveness, and 55 analyses of intervention effectiveness (Additional file 2 Tables S7 to S14). The dates of published studies included in the reviews examined ranged from 1966 to 2008.

### Study quality

The methodological quality of included reviews (Additional file 1 Tables S4, S6) was generally good (median OQAQ score = 15.6). The most common methodological weaknesses were the lack of use of study quality data to inform analyses (e.g. by sensitivity analysis, or by constructing separate analyses which excluded low quality trials) and potential bias in the selection of articles (e.g. not using independent assessors).

### Evidence synthesis

The extracted analyses and evidence grades for each analysis are presented in Additional file 2 Tables S7 to S14. The findings can be summarised as follows:-

### Overall effectiveness (Additional file 2, Table S7)

#### Weight Loss

High quality causal evidence (grade 1++) from eight meta-analyses of RCTs from four reviews showed that interventions to promote changes in diet (or both diet and physical activity) produced moderate and clinically meaningful effects on weight loss (typically 3-5 kg at 12 months, 2-3 kg at 36 months)[37,38,42,50]. The effectiveness of such interventions (as well as physical activity only interventions) in producing weight loss was further supported by medium and low quality causal evidence (grade 1+ and 1-) from 14 meta-analyses and summaries of RCTs from six reviews (eight medium, six low quality analyses)[31,39,49,54,57,59].

#### Physical Activity

High quality causal evidence was found from four meta-analyses of RCTs in two reviews that physical activity interventions can produce moderate changes in self-reported physical activity (standardised mean difference around 0.3; Odds Ratio for achieving healthy activity targets around 1.2 to 1.3) and cardio-respiratory fitness (standardised mean difference around 0.5) at a minimum 6 months of follow up[41,59]. This was supported by lower quality causal evidence from six meta-analyses of RCTs and summaries of RCTs and other studies (three medium and three low quality analyses) from three systematic reviews that interventions to increase physical activity increased self-reported physical activity (typically equivalent to 30-60 minutes of walking per week) at a median of 6 weeks to 19 months of follow up[38,40,51]. However, it is worth noting that there were few examples of trials with successful outcomes at more than 12 months.

#### Dietary Intake

Medium and lower quality causal evidence from meta-analyses and descriptive summaries of RCTs (nine analyses from three separate reviews: six medium, three low) that found positive changes in self-reported diet (calorie, fat, fibre, fruit and vegetable intake) at 6 to 19 months of follow up for dietary interventions[38,34,44].

#### Other Outcomes

High quality causal evidence (grade 1++) from one meta-analysis of RCTs[43] showed that interventions to promote changes in diet or physical activity (or both) produced moderate and clinically meaningful effects on the risk of progression to type 2 diabetes (relative risk reduction of 49% at 3.4 years) in people with impaired glucose regulation.

One review which examined variations in effectiveness over time[37] showed that weight loss tended to reverse once interventions ceased or moved from an active to a maintenance phase (net weight loss during active phase 0.08 BMI units per month; net weight *gain *during maintenance phase 0.03 BMI units per month).

### Theoretical basis (Additional file 2, Table S8)

One meta-regression analysis provided medium quality associative evidence (grade 2+) suggesting that interventions with an explicitly stated theoretical basis (e.g. Social Cognitive Theory,[60] Theory of Planned Behaviour[61]) were no more effective in producing changes in either weight or in combined dietary and physical activity outcomes than interventions with no stated theoretical basis[38]. However, four meta-regression analyses (all medium quality associative analyses) in two reviews[38,48] did find an association between the use of a theoretically specified cluster of 'self-regulatory' intervention techniques (specific goal-setting, prompting self-monitoring, providing feedback on performance, goal review) and increased effectiveness in terms of a) weight loss, b) change in dietary outcomes, c) change in physical activity and d) combined (standardised mean difference for either dietary change or physical activity) outcomes.

### Behaviour change techniques (Additional file 2, Table S9)

Categorisation of interventions varied greatly between reviews, with categories often conceptually overlapping and vaguely defined (e.g. diet vs. exercise vs. behavioural intervention). Despite this, we have summarised evidence on the use of what we have called "established, well defined behaviour change techniques", based on those reviews where clear and specific definitions were provided (see Table 1 for definitions). Further definition of the specific behaviour change techniques cited in Table 1 and those mentioned in the text below can be found in a recent taxonomy of behaviour change techniques[62].

Causal evidence from one medium quality meta-analysis indicated that change in weight was greater when established, well defined behaviour change techniques were added to interventions (e.g. when dietary advice plus a well-defined behavioural intervention using established behaviour change techniques was compared with dietary advice alone). The weight loss achieved by adding established behaviour change techniques to interventions was 4.5 kg at a median 6 months of follow up[54]. This was supported by two associative analyses (one medium and one low quality) which compared the results of different groups of studies in which the interventions either did or did not use established, well-defined behaviour change techniques. Using established behaviour change techniques was associated with increased weight loss (2.5 to 5.5 kg) compared with non-behavioural interventions (0.1 to 0.9 kg)[46,47].

Evidence from five low to medium quality associative analyses in two reviews attempted to relate the number of behaviour change techniques used to effectiveness in terms of weight loss or changes in diet or physical activity. The evidence was equivocal with the pattern of data suggesting a possible association, but only one analysis approached significance[38,48].

#### Use of specific behaviour change techniques

High quality causal evidence was found that adding social support to interventions (usually from family members) provided an additional weight loss of 3.0 kg at up to 12 months (compared with the same intervention with no social support element)[31].

Medium to low quality associative evidence (from three meta-regression analyses and two associative analyses in three reviews) suggested that effectiveness for initial behaviour change (i.e. change in weight, diet or physical activity was associated with using the following techniques (NB: definitions of these can be found in a recent taxonomy of behaviour change techniques[62]): 1) For dietary change: providing instruction, establishing self-monitoring of behaviour, use of relapse prevention techniques[38,48]. 2) For physical activity change: prompting practice, establishing self-monitoring of behaviour, individual tailoring (e.g. of information or counselling content)[38,40,48]. One review also provided medium quality causal evidence (a descriptive summary of individual RCT findings) that brief advice, which usually included goal-setting, led to an increase in walking activity (27 mins/week walking at 12 months of follow up)[51]. Goal-setting alongside the use of pedometers was also associated with increased walking (see below).

Further medium quality associative evidence suggested that increased *maintenance *of behaviour change was associated with the use of time management techniques (for physical activity) and encouraging self-talk (for both dietary change and physical activity)[38].

Three reviews examined interventions that used pedometers (i.e. self-monitoring of physical activity) to promote walking: Medium quality causal evidence (two analyses from two reviews) supported the effectiveness of pedometer based interventions for increasing walking activity[33,51] (mean increase of 2004 steps per day at a median 11 weeks; median increase in time walking of +54 min per week at a median 13 weeks). It must be noted that the vast majority of the interventions included in these meta-analyses included either step-goals or step diaries (or both) alongside the use of pedometers, so the evidence does not support the use of pedometers in isolation from these additional techniques. Indeed, associative analyses from one review[33] suggested that the use of a) a step diary (one low quality analysis) and b) goal-setting (one low and one medium quality analysis) in combination with use of a pedometer was associated with increased walking. Medium to high quality associative evidence (based on meta-analysis of only the intervention arms of studies) from two reviews[33,52] suggested that small changes in weight might also be achievable with pedometer based interventions (e.g. change in BMI of 0.38 kg/m2 at 11 weeks).

#### Motivational interviewing

Motivational interviewing is a distinct combination of behaviour change techniques (including decisional balance and relapse prevention techniques) delivered in a specific style (using patient centred empathy building techniques, such as rolling with resistance; affirmation and reflective listening)[63]. High quality causal evidence from one meta-analysis of RCTs[53] found that motivational interviewing was significantly more effective than traditional advice-giving for initiating changes in weight (producing a net difference of 0.72 BMI units compared with traditional advice-giving) at 3 to 24 months of follow up (mostly under 6 months). A further meta-analysis of RCTs[35] provided medium quality causal evidence of the effectiveness of motivational interviewing for a combined physical activity and dietary outcome, at up to 4 months of follow up (Standardised Mean Difference 0.53).

#### Targeting multiple behaviours

Causal evidence from nine analyses in four reviews (one high, four medium and four low quality) showed that interventions which targeted *both *physical activity and diet rather than only one of these behaviours produced higher weight change (additional weight loss around 2-3 kg at up to 12 months)[31,36,37,54].

### Mode of delivery (Additional file 2, Table S10)

The evidence from five reviews of dietary and/or physical activity intervention was mixed. Five associative analyses (three medium and two low quality) from four reviews failed to find a clear association between effectiveness and mode of intervention delivery for weight loss, dietary change or physical activity change[38,46,48,51]. One review found medium quality associative evidence that 'mixed mode' (individual and group) delivery was significantly related to greater effectiveness, compared with individual delivery, for initial weight loss (up to 6 months), but not for weight loss maintenance (at a mean 19 months)[38]. However, it is worth noting that there is evidence from individual high quality RCTs (based on data in the evidence tables of the included reviews) that individual, group, and mixed mode interventions can all be effective in changing diet and/or physical activity[31,38,51].

### Intervention provider (Additional file 2, Table S11)

There was a lack of high quality evidence in this area for comparisons between specific types of intervention provider. Four associative analyses (two medium, two low) from four reviews provided no consistent or significant relationship between intervention provider and weight, physical activity or dietary outcomes at up to 12 months of follow up[38,40,48,51]. However, strong evidence from individual RCTs (based on data in the evidence tables of the included reviews) showed that a wide range of providers (with appropriate training) including doctors, nurses, dieticians/nutritionists, exercise specialists and lay people, can deliver effective interventions for changing diet and/or physical activity[38,40,43,48,51,52].

### Intervention intensity (Additional file 2, Table S12)

Definitions of intervention intensity reported in the reviews varied considerably, incorporating frequency and total number of contacts, total contact time, duration of the intervention and the number of behaviour change techniques used. The frequency and duration of clinical contact varied widely, ranging from 1 to around 80 sessions, delivered daily to monthly and lasting anything from 15 to 150 minutes, over periods ranging from 1 day to 2 years. For instance, one review of 17 weight loss interventions that compared different intervention intensities, reported that the median contact frequency was weekly, the median session duration 60 minutes, and the median delivery period 10 weeks[54]. Physical activity interventions are often much more intensive due to a focus on practising the target behaviour (e.g. Shaw et al.[55] report interventions lasting 3 to 12 months with 3 to 5 sessions per week lasting a median 45 minutes each).

#### Weight Loss

Overall, 7 out of 9 analyses of intervention intensity favoured higher intensity interventions. One meta-analysis of ten small RCTs (N = 306) comparing different intervention intensities[54] found medium quality causal evidence that more intensive interventions (those including more behaviour change techniques, more contact time or a longer duration of intervention) generated significantly more weight loss than less intensive interventions (an additional 2.3 kg at a median seven months follow up). This was supported by a medium quality associative analysis from the same review. However, it was not possible to deduce from the available data which component of intensity drives this relationship.

Medium to low quality evidence from three analyses in three reviews (one medium quality, two low quality) showed a positive association between the *total number of contacts *and weight loss at 12 to 38 months[46,50,57]. Associative evidence from two analyses in two reviews (one high quality, one low quality) found a relationship between *increased frequency of contacts *and weight loss at 6 to 15 months of follow up[37,47]. However, two associative analyses (one high and one medium quality) in two reviews[37,38] found no such relationship at 6 to 60 months. Two medium quality associative analyses found mixed evidence (one positive one negative) on the association between intervention *duration *and weight loss.

#### Dietary Change

Two low quality associative analyses within the same review found a positive relationship between number of contacts and self-reported dietary change at 12 months of follow up[34].

#### Physical Activity

There was a lack of evidence on the relationship between intervention intensity and physical activity outcomes. Two low quality associative analyses in two reviews[33,40] found no clear relationship between intervention intensity (duration) and physical activity outcomes.

### Characteristics of the target population (Additional file 2, Table S13)

#### Gender

Eight associative analyses (three medium quality, five low quality) from six reviews found no consistent association between gender and changes in weight or physical activity at 10 weeks to 16 months of follow up[33,38,41,48,55,58].

#### Ethnicity

Although there is evidence (within some of the component trials in the reviews examined) that interventions can be effective for a number of ethnic groups[4] there was very little review-level evidence on the relationship between ethnicity and intervention effectiveness. One associative analysis (low quality) suggested that intervention studies with a higher percentage of white Caucasian participants achieved larger decreases in BMI at a median of 12 weeks of follow up[33]. Another (low quality) associative analysis in the same review reported no association between ethnicity and increased walking.

#### Age

Associative analyses (one medium quality, one low quality) from two reviews[33,55] suggested that older people lost more weight than younger people at 10.5 to 16 weeks of follow up[33]. Two further (low quality) analyses from two reviews found no relationship between age and physical activity at 3 and 6 months of follow up[33,41].

#### At risk populations

A range of evidence, including strong causal evidence from two meta-analyses of sub-groups of studies and associative evidence from meta-regression analyses from several further reviews found that changes in weight and (at least short-term) physical activity are possible in high risk as well as lower risk populations, including high and low weight, high cardiovascular risk groups and sedentary and non-sedentary groups, at between 3 and 36 months of follow up[33,37,38,41-43,48,51]. Five analyses from four reviews provided mixed evidence as to whether targeting of interventions at people who are more sedentary was associated with larger increases in the amount of physical activity (two medium analyses (one positive, one negative), three low quality analyses (two negative, one trend)[33,41,48,51].

#### Diabetes

In two associative analyses (one high quality, one medium quality), effectiveness for weight loss (at 3 to 60 months) was found to be considerably lower for people with type 2 diabetes than for people without type 2 diabetes[37,38].

#### Weight

Four analyses in four reviews[33,41,42,48] provided mixed associative evidence (two medium (one positive, one negative), two low quality analyses (one positive, one negative)) as to whether targeting more overweight people was associated with larger increases in the amount of weight loss achieved. However, one high quality associative analysis showed that people with a higher starting weight achieve better *health *improvements at 2 to 4.6 years, in terms of a reduced incidence of type 2 diabetes[43].

### Setting (Additional file 2, Table S14)

Examples were found (based on data in the evidence tables of included reviews) of effective interventions delivered in a wide range of settings, including healthcare settings, the workplace, the home, and in the community[30,34]. Few reviews formally examined the impact of intervention setting on effectiveness. However, one medium quality associative analysis revealed no significant differences in outcomes (either dietary or physical activity change) at six months between interventions in primary care, community and workplace settings[48].

## Discussion

This review has, for the first time, systematically identified, synthesised *and graded *a wide range of evidence about the relationship of intervention content to effectiveness in individual-level interventions for promoting changes in diet and/or physical activity in adults at risk of type 2 diabetes.

Interventions produced significant and clinically meaningful changes in physical activity (typically equivalent to 30-60 minutes of walking per week, for up to 18 months) and in weight (typically 3-5 kg at 12 months, 2-3 kg at 36 months). Greater effectiveness of interventions was causally linked (in meta-analyses and randomised trials which experimentally manipulated the use of these elements) with targeting both diet and physical activity, mobilising social support and the use of well-described/established behaviour change techniques. Greater effectiveness was also associated (in correlational analyses and non-randomised comparisons) with using a cluster of self-regulatory techniques (goal-setting, prompting self-monitoring, providing feedback on performance, goal review[62,64]), and providing a higher contact time or frequency of contacts. However, with regard to intensity, the amount of clinical contact in interventions varied widely (see ranges reported above) and the evidence did not support the recommendation of any particular minimum threshold. The evidence on patterns of effectiveness over time[37] also suggested that there is a need for an increased focus on the use of techniques to support behaviour maintenance.

There were no clear associations between provider, setting, delivery mode, ethnicity and age of the target group and effectiveness. This (and evidence from a range of individual RCTs cited in the reviews examined) suggests that interventions can be delivered successfully by a wide range of providers in a wide range of settings, in group or individual or combined modes, and can be effective for a wide range of ethnic and age groups.

While the use of "established, well-defined behaviour change techniques" was associated with increased effectiveness, it is worth emphasising that individual techniques are rarely applied in isolation and should form part of a coherent intervention model. Therefore, a planned approach to intervention design is recommended, such as "intervention mapping",[65] or other systematic intervention development processes[66] which select intervention techniques to address targeted behaviour change processes (and that are tailored for the target population and setting).

Taken together, the findings suggest a number of recommendations for optimising practice in the development and delivery of interventions to promote changes in diet and/or physical activity and these are outlined in Table 2. It is hoped that applying these findings will help to meet the growing need for less costly, but nonetheless effective, type 2 diabetes prevention programmes.

**Table 2:** Recommendations for practice

A^1^	Interventions should aim to promote changes in both diet and physical activity
**A**	Interventions should use established, well defined behaviour change techniques (e.g. Specific goal-setting, relapse prevention, self-monitoring, see Table 1)

**A**	Interventions should encourage participants to engage social support in planned behaviour change (i.e. engage others who are important such as family, friends, and colleagues)

**A**	Interventions may be delivered by a wide range of people/professions, subject to appropriate training. There are examples of successful physical activity and/or dietary interventions delivered by doctors, nurses, dieticians/nutritionists, exercise specialists and lay people, often working within a multi-disciplinary team

**A**	Interventions may be delivered in a wide range of settings. There are examples of successful physical activity and/or dietary interventions delivered in healthcare settings, the workplace, the home, and in the community

**A**	Interventions may be delivered using group, individual or mixed modes (individual and group). There are examples of successful physical activity and/or dietary interventions using each of these delivery modes

**A**	Interventions should include a strong focus on maintenance. It is not clear how best to achieve behaviour maintenance but behaviour change techniques designed to address maintenance include: self-monitoring of progress, providing feedback, reviewing of goals, engaging social support, use of relapse management techniques and providing follow-up prompts

**B**	Interventions should maximise the frequency or number of contacts with participants

**C**	Interventions may consider building on a coherent set of "self-regulation" techniques, which have been associated with increased effectiveness (Specific goal setting; Prompting self-monitoring; Providing feedback on performance; Review of behavioural goals) as a starting point for intervention design. However, this is not the only approach available

**C**	No specific intervention adaptations are recommended for men or women, although it may be important to take steps to increase engagement and recruitment of men

**D**	If using established behaviour change techniques, a clear plan of intervention should be developed, based on a systematic analysis of factors preceding, enabling and supporting behaviour change in the social/organisational context in which the intervention is to be delivered. The plan should identify the processes of change and the specific techniques and method of delivery designed to achieve these processes. Such planning should ensure that the behaviour change techniques and strategies used are mutually compatible and well-adapted to the local delivery context. Following the procedures of the PRECEDE-PROCEED model [62], Intervention Mapping [61], or a similar intervention-design procedure is recommended

**D**	People planning and delivering interventions should consider whether adaptations are needed for different ethnic groups (particularly with regard to culturally-specific dietary advice), people with physical limitations and people with mental health problems

^1^Key to grades of recommendations:

A: At least one meta-analysis, systematic review, or RCT rated as 1++ and directly applicable to the target population; *or *A body of evidence

consisting principally of studies rated as 1+, directly applicable to the target population, and demonstrating overall consistency of results

B: A body of evidence including studies rated as 2++, directly applicable to the target population, and demonstrating overall consistency of

results; *or *Extrapolated evidence from studies rated as 1++ or 1+

C: A body of evidence including studies rated as 2+, directly applicable to the target population and demonstrating overall consistency of results;

*or *Extrapolated evidence from studies rated as 2++

D: Evidence level 3 or 4 (non-analytic studies or expert opinion)*; or E*xtrapolated evidence from studies rated as 2+

Although providing a greater degree of depth with regard to intervention components, these findings are consistent with UK guidance for the prevention and treatment of obesity (which recommends engaging social (especially family based) support, and targeting both diet and exercise)[67]. The findings are also consistent with recent guidance from the American Heart Association[68] on the prevention of heart disease in adults aged over 18, which recommend the use of motivational interviewing as well as goal-setting, self-monitoring and a high contact frequency. Recent evidence-based guidance from the US Association of Diabetes Educators also recommends goal-setting, problem-solving (relapse prevention) and self-monitoring of plans (self-regulation) for supporting healthy eating and increased physical activity in people with type 2 diabetes[69]. Our findings may also be more widely generalisable to adults with diagnosed chronic disease (e.g. type 2 diabetes, heart disease) or to apparently healthy adults.

### Strengths and limitations

Our review focused only on higher quality systematic reviews. We identified a substantial number of reviews which synthesised data from a large number of RCTs and other studies, in a wide range of age groups, clinical/risk groups and settings. Drawing together these findings in one place has generated a comprehensive, evidence-based overview of which intervention components are most likely to facilitate effectiveness.

However, several challenges affecting the synthesis and interpretation of the available evidence were encountered. One of the limitations most commonly cited by review authors was an inadequate description of behavioural interventions in the individual study reports. This causes difficulties for the reviewer in categorising intervention content and conducting subsequent analyses to relate content to effectiveness. We therefore suggest that future intervention study reports (and reviews of individual studies) use an appropriate taxonomy to describe (and categorise) behaviour change techniques[62]. A major limitation in assessing the utility of specific theories and techniques underpinning interventions is that techniques may not be implemented rigorously or may not faithfully represent the specified theories[62,70]. Notably, none of the 30 reviews that we examined took intervention fidelity into account. Hence, the lack of an association between the use of a stated theory and effectiveness may reflect a lack of good theories or it may reflect poor implementation of theories. Other potentially important sources of bias include measurement issues (especially in relation to the use of self-report data); self-selection of intervention participants; and a failure to consider potential biases due to study quality in some reviews. Furthermore, it is worth noting that with associative evidence, other covariates than those analysed may account for the stated relationships (e.g. the association between intensity and effectiveness might be explained to some extent by lower quality of intervention being associated with lower intensity).

A further potential source of bias which no review accounted for was the low sample size contributing to some of the analyses examined. In particular, it is worth noting that, whilst our recommendation (Table 2) on the usefulness of social support technically merits a grade A (as it is based on level 1+ evidence from a meta-analysis of randomised controlled trials), the total number of participants contributing to the meta-analysis was only 127. If the grading system had taken sample size into account, we may have given this recommendation a lower grade. In interpreting the above information, it should be noted that the analyses considered were in many cases based on overlapping sets of trials (and other studies). It should also be noted, as this is a review of reviews we were not able to synthesise or meta-analyse data from individual studies, which may have yielded valuable evidence. It is also worth noting that at the time of the literature search there were no high quality reviews on the use of internet-based interventions, so no evidence is presented in this area.

### Implications for practice and policy

Our review has generated clear recommendations on how interventions for promoting lifestyle change within diabetes prevention programmes could be developed or refined to maximise effectiveness (Table 2). Our recommendations go considerably beyond the data on basic effectiveness presented in trials and systematic reviews of diabetes prevention programmes to date[3-8]. They can be useful, for example, in guiding the translation of effective, high-intensity/high resource-use interventions in research contexts into lower-cost (yet still effective) interventions for implementation in clinical practice.

### Directions for future research

More rigorous evaluations of the effectiveness and cost-effectiveness of specific intervention components and clusters of techniques for promoting and maintaining change in diet and physical activity are needed. This will require experimental and theoretically driven manipulation of intervention components in well-powered and high-quality trials. Intervention studies need to provide careful descriptions of the hypothesised causal processes for achieving behaviour change and the specific techniques used to modify these processes. Trials should include process analyses to establish the validity or otherwise of the causal models proposed. Research is urgently needed to compare the cost-effectiveness of interventions with different providers, intervention modes and intensities (using clear and consistent conceptualisations of intensity and attempting to disentangle the different elements of intensity such as contact time, number of contacts and contact frequency). This should include the evaluation of remotely delivered and/or self-delivered (e.g. internet-based) approaches and other approaches that might provide high effectiveness for lower cost. Research is also needed to establish the impact of the intervention setting on effectiveness; to optimise intervention procedures for different ethnic, age and gender groups; to establish effective techniques for improving recruitment to interventions (and to address gender imbalances); and to assess the possible adverse affects of dietary and physical activity interventions.

## Conclusions

Interventions to promote changes in diet and/or physical activity in adults with increased risk of diabetes or cardiovascular disease are more likely to be effective if they a) target both diet and physical activity, b) involve the planned use of established behaviour change techniques, c) mobilise social support, and d) have a clear plan for supporting maintenance of behaviour change. They may also benefit from providing a higher frequency or total number of contacts.

To maximise the effectiveness of intervention programmes to promote changes in diet and/or physical activity for diabetes prevention, practitioners and commissioning organisations should carefully consider the inclusion of the above components.

## Competing interests

The authors declare that they have no competing interests.

## Authors' contributions

CG conceived and coordinated the study. KS and CG conducted literature searches, data extraction, review selection, quality rating and evidence grading and drafted the manuscript. CA, WH, MR, PE and PS contributed to the design of the study and interpretation of the results. All authors read and approved the final manuscript.

## Pre-publication history

The pre-publication history for this paper can be accessed here:

http://www.biomedcentral.com/1471-2458/11/119/prepub

## Supplementary Material

Additional file 1 **Table S1: Search Strategy**. Table S2 (and explanatory text): OQAQ: Quality assessment tool for systematic reviews and meta-analyses. Table S3 (and explanatory text): Evidence Grading System. Table S4: Characteristics of Included Reviews. Table S5: Excluded papers. Table S6: OQAQ scores. Click here for file

Additional file 2 **Tables S7-14: Data from analyses of: S7) Intervention Effectiveness; S8) Theoretical basis; S9) Behaviour change techniques; S10) Mode of delivery; S11) Intervention provider; S12) Intervention intensity; S13) Intervention population; S14) Intervention setting**. Click here for file

Additional file 3 **PRISMA (Preferred Reporting Items for Systematic Reviews and Meta-Analyses) 2009 Checklist**. Click here for file
