# Supplementary material for: Systematic review of reviews of intervention components associated with increased effectiveness in dietary and physical activity interventions
Source: BMC Public Health. 2011 Feb 18;11:119. doi: 10.1186/1471-2458-11-119 (PMC3048531; doi:10.1186/1471-2458-11-119)
Supplement: Additional file 1 — Table S1: Search Strategy. Table S2 (and explanatory text): OQAQ: Quality assessment tool for systematic reviews and meta-analyses. Table S3 (and explanatory text): Evidence Grading System. Table S4: Characteristics of Included Reviews. Table S5: Excluded papers. Table S6: OQAQ scores. [file 1471-2458-11-119-S1.DOC]

**Additional file 1: Supplementary Tables S1 to S6**

Table S1: Search Strategy. Table S2 (and explanatory text): OQAQ: Quality assessment tool for systematic reviews and meta-analyses. Table S3 (and explanatory text): Evidence Grading System. Table S4: Characteristics of Included Reviews. Table S5: Excluded papers. Table S6: OQAQ scores.

**Table S1: Search strategy**

Unless otherwise stated, search terms were free text terms; MeSH terms: Medical subject heading (MEDLINE medical index term); the dollar sign ($) stands for any character and a number directly after a dollar sign denotes the maximum number of additional letters after the word-stem. The search strategy for MEDLINE is presented below. This strategy was adapted for each database used.

| 1. | (RISK NEAR DIABETES).TI,AB. |
| --- | --- |
| 2. | (RISK NEAR HEART).TI,AB. |
| 3. | (RISK NEAR CARDIOVASCULAR).TI,AB. |
| 4. | (RISK NEAR CVD).TI,AB. |
| 5. | (CARDIOVASCULAR ADJ RISK).TI,AB. |
| 6. | (RISK ADJ CARDIOVASCULAR).TI,AB. |
| 7. | SEDENTARY.TI,AB. |
| 8. | OBESITY.W.DE. OR DIABETES-MELLITUS-TYPE-2.DE. OR HYPERTENSION.W.DE. OR OVERWEIGHT.W.DE. |
| 9. | INACTIVE.TI,AB. |
| 10. | OVERWEIGHT.TI,AB. |
| 11. | (OVER ADJ WEIGHT).TI,AB. |
| 12. | OBES$3.TI,AB. |
| 13. | OBESITY-MORBID.DE. |
| 14. | DIABET$2.TI,AB. |
| 15. | HYPERTENS$3.TI,AB. |
| 16. | (HIGH ADJ BLOOD ADJ PRESSURE).TI,AB. |
| 17. | (GLUCOSE ADJ INTOLERANC$3).TI,AB. |
| 18. | (IMPAIRED ADJ GLUCOSE ADJ TOLERANC$3).TI,AB. |
| 19. | (IMPAIRED ADJ FASTING ADJ GLUCOSE).TI,AB. |
| 20. | HYPERLIPID$5.TI,AB. |
| 21. | HYPERGLYC$5.TI,AB. |
| 22. | (METABOLIC ADJ SYNDROME).TI,AB. |
| 23. | (HIGH ADJ CHOLESTEROL).TI,AB. |
| 24. | HYPERCHOLESTEROL$5.TI,AB. |
| 25. | (FAMILIAL ADJ HYPERLIPID$5).TI,AB. |
| 26. | PREDIABETES.TI,AB. |
| 27. | (PRE ADJ DIABETES).TI,AB. |
| 28. | (POLYCYSTIC ADJ OVARIAN ADJ SYNDROME).TI,AB. |
| 29. | (FAMILY ADJ HISTORY).TI,AB. |
| 30. | (GESTATIONAL ADJ DIABETES).TI,AB. |
| 31. | (METABOLIC ADJ SYNDROME).TI,AB. |
| 32. | METABOLIC-SYNDROME-X.DE. OR POLYCYSTIC-OVARY- SYNDROME.DE. |
| 33. | PREDIABETIC-STATE.DE. OR GLUCOSE-INTOLERANCE.DE. |
| 34. | HYPERCHOLESTEROLEMIA.W.DE. OR HYPERLIPIDEMIAS.W.DE. |
| 35. | DIABETES-GESTATIONAL.DE. OR HYPERGLYCEMIA.W.DE. |
| 36. | HYPERLIPIDEMIA-FAMILIAL-COMBINED.DE. |
| 37. | 1 OR 2 OR 3 OR 4 OR 5 OR 6 OR 7 OR 8 OR 9 OR 10 OR 11 OR 12 OR 13 OR 14 OR 15 OR 16 OR 17 OR 18 OR 19 OR 20 OR 21 OR 22 OR 23 OR 24 OR 25 OR 26 OR 27 OR 28 OR 29 OR 30 OR 31 OR 32 OR 33 OR 34 OR 35 OR 36 |
| 38. | OBESITY.W.DE. OR HYPERTENSION.W.DE. OR OVERWEIGHT.W.DE. |
| 39. | 1 OR 2 OR 3 OR 4 OR 5 OR 6 OR 7 OR 9 OR 10 OR 11 OR 12 OR 13 OR 15 OR 16 OR 17 OR 18 OR 19 OR 20 OR 21 OR 22 OR 23 OR24 OR 25 OR 26 OR 27 OR 28 OR 29 OR 30 OR 31 OR 32 OR33 OR34 OR 35 OR 36 OR 38 |
| 40. | PREVENT$5.TI,AB. |
| 41. | (PATIENT ADJ EDUCATION).TI,AB. |
| 42. | PATIENT-EDUCATION-AS-TOPIC.DE. OR SELF-CARE.DE. |
| 43. | (PRIMARY ADJ PREVENTION).TI,AB. |
| 44. | HEALTH-PROMOTION.DE. OR DIET.W.DE. OR FOOD-HABITS.DE. |
| 45. | (HEALTH$3 ADJ BEHAV$6).TI,AB. |
| 46. | HEALTH-BEHAVIOR.DE. OR LIFE-STYLE.DE. OR WALKING.W.DE. OR HEALTH-EDUCATION.DE. OR RISK-REDUCTION-BEHAVIOR.DE. |
| 47. | (HEALTH$3 ADJ EDUCAT$5).TI,AB. |
| 48. | COUNSELING.W.DE. |
| 49. | (HEALTH$3 ADJ PROMOT$5).TI,AB. |
| 50. | EXERCISE.W.DE. OR PHYSICAL-FITNESS.DE. OR SPORTS.W.DE. |
| 51. | MOTIVAT$5.TI,AB. |
| 52. | (SELF ADJ MANAGEMENT).TI,AB. |
| 53. | PATIENT-CENTERED-CARE.DE. |
| 54. | (SELF ADJ CARE).TI,AB. |
| 55. | (SELF ADJ REGULATION).TI,AB. |
| 56. | (PROBLEM ADJ SOLVING).TI,AB. |
| 57. | PROBLEM-SOLVING.DE. |
| 58. | (PERSON ADJ CENTRED).TI,AB. |
| 59. | (PERSON ADJ CENTERED).TI,AB. |
| 60. | (CLIENT ADJ CENTERED).TI,AB. |
| 61. | (CLIENT ADJ CENTRED).TI,AB. |
| 62. | (PATIENT ADJ CENTERED).TI,AB. |
| 63. | (PATIENT ADJ CENTRED).TI,AB. |
| 64. | DIET.TI,AB. |
| 65. | (BEHAV$5 ADJ THEOR$3).TI,AB. |
| 66. | (BEHAV$5 ADJ THEOR$3).TI,AB. |
| 67. | PSYCHOLOGICAL-THEORY.DE. OR BEHAVIOR-THERAPY.DE. OR COGNITIVE-THERAPY.DE. |
| 68. | (AEROBIC ADJ TRAIN$3).TI,AB. |
| 69. | EXERCISE-THERAPY.DE. OR PHYSICAL-ENDURANCE.DE. OR PHYSICAL-EDUCATION-AND-TRAINING.DE. OR SWIMMING.W.DE. OR BICYCLING.W.DE. |
| 70. | (STRENGTH ADJ TRAIN$3).TI,AB. |
| 71. | WEIGHT-LOSS.DE. |
| 72. | (RESISTANCE ADJ TRAIN$3).TI,AB. |
| 73. | WALK$3.TI,AB. |
| 74. | BICYCL$3.TI,AB. |
| 75. | SWIM$4.TI,AB. |
| 76. | SPORT$1.TI,AB. |
| 77. | FITNESS.TI,AB. |
| 78. | EXERCIS$3.TI,AB. |
| 79. | WEIGHT.TI,AB. |
| 80. | LIFESTYLE.TI,AB. |
| 81. | (LIFE ADJ STYLE).TI,AB. |
| 82. | BEHAV$6.TI,AB. |
| 83. | NUTRITION.TI,AB. |
| 84. | COUNSEL$5.TI,AB. |
| 85. | (PHYSICAL ADJ FITNESS).TI,AB. |
| 86. | (PHYSICAL$2 ADJ ACTIV$5).TI,AB. |
| 87. | BODY-WEIGHT.DE. OR WEIGHT-GAIN.DE. OR DIET- REDUCING.DE. |
| 88. | (PHYSICAL ADJ ENDURANCE).TI,AB. |
| 89. | ENERGY-INTAKE.DE. |
| 90. | (HEALTH$3 ADJ BEHAV$6).TI,AB. |
| 91. | HEALTH-BEHAVIOR.DE. OR FOOD-HABITS.DE. |
| 92. | (PSYCHOLOGICAL ADJ THEORY).TI,AB. |
| 93. | (AEROBIC ADJ TRAIN$3).TI,AB. |
| 94. | INTERVENTION.TI,AB. |
| 95. | WALKING.W.DE. |
| 96. | 40 OR 41 OR 42 OR 43 OR 44 OR 45 OR 46 OR 47 OR 48 OR 49 OR 50 OR 51 OR 52 OR 53 OR 54 OR 55 OR 56 OR 57 OR 58 OR 59 OR 60 OR 61 OR 62 OR 63 OR 64 OR 65 OR 66 OR 67 OR 68 OR 69 OR 70 OR 71 OR 72 OR 73 OR 75 OR 76 OR 77 OR 78 OR 79 OR 80 OR 81 OR 82 OR 83 OR 84 OR 85 OR 86 OR 87 OR 88 OR 89 OR 90 OR 91 OR 92 OR 93 OR 94 OR 95 |
| 97. | WEIGHT.TI,AB. |
| 98. | (PHYSICAL$2 ADJ ACTIV$5).TI,AB. |
| 99. | EXERCIS$3.TI,AB. |
| 100. | WALK$3.TI,AB. |
| 101. | EXERTION$2.TI,AB. |
| 102. | (ENERGY ADJ EXPENDITURE).TI,AB. |
| 103. | BMI.TI,AB. |
| 104. | (BODY ADJ MASS ADJ INDEX).TI,AB. |
| 105. | (WAIST ADJ CIRCUMFERENCE).TI,AB. |
| 106. | (WAIST ADJ TO ADJ HIP).TI,AB. |
| 107. | 97 OR 98 OR 99 OR 100 OR 101 OR 102 OR 103 OR 104 OR 105 OR 106 |
| 108. | (SYSTEMATIC ADJ REVIEW).TI,AB. |
| 109. | REVIEW.TI,AB. |
| 110. | (META ADJ ANALYSIS).TI,AB. |
| 111. | META-ANALYSIS.TI,AB. |
| 112. | GUIDELINE$1.TI,AB. |
| 113. | REVIEW=YES |
| 114. | PT=META-ANALYSIS |
| 115. | METAANALYSIS.TI,AB. |
| 116. | (SYSTEMATIC NEAR (REVIEW$3 OR OVERVIEW)).TI,AB. |
| 117. | (QUANTITATIV$2 NEAR (REVIEW$3 OR OVERVIEW$2 OR SYNTHESIS$2)).TI,AB. |
| 118. | 108 OR 109 OR 110 OR 111 OR 112 OR 113 OR 114 OR 115 OR 116 OR 117 |
| 119. | OBSERVATIONAL.TI,AB. |
| 120. | RCT.TI,AB. |
| 121. | INTERVENTION$1.TI,AB. |
| 122. | (RANDOMI$4 ADJ CONTROL ADJ TRIAL$1).TI,AB. |
| 123. | (QUASI ADJ EXPERIMENTAL).TI,AB. |
| 124. | TRIAL$1.TI,AB. |
| 125. | 119 OR 120 OR 121 OR 122 OR 123 OR 124 |
| 126. | 37 AND 96 AND 118 AND 125 |
| 127. | 39 AND 96 AND 118 AND 125 |
| 128. | 126 AND 107 |
| 129. | 127 AND 107 |
| 130. | 128 NOT 129 |
| 131. | 129 AND (CHILD# OR ADOLESCENT.DE. OR INFANT#) |
| 132. | 129 AND ANIMAL=YES |
| 133. | 129 NOT 131 |
| 134. | 133 NOT 132 |

**Table S2: OQAQ: Quality assessment tool for systematic reviews and meta-analyses**

A modified version of the OQAQ was used to assess the quality of reviews. This consists of the following nine questions each answerable as ‘yes’, ‘no’ or ‘partially/can’t tell’, carrying scores of 2, 0 and 1, respectively.

Quality Criteria: Reviews were included if their OQAQ score was 14 or more (possible range: 0-18) and if they met at least one of the two OQAQ criteria (scored minimum 1 point on either question 5 &/or 6) about assessing study quality and taking quality into account in analyses (this emphasis on study quality was intended to maximise the likely quality of evidence underlying the review-level analyses).

| 1. **Were the search methods used to find evidence on the primary question(s) stated?** |
| --- |
| 1. **Yes**, description of databases searched, search strategy, and years reviewed. **2 points**. |
| 1. **Partially**, descriptions of methods not complete. **1 point**. |
| 1. **No**, no description of search methods. **0 points**. |
| 1. **Was the search for evidence reasonably comprehensive?** |
| 1. **Yes**, at least one computerised database searched and also a search of unpublished or non-indexed literature. **2 points**. |
| 1. **Can’t tell**, search strategy partially comprehensive, at least one of the strategies performed. **1 point**. |
| 1. **No**, search not comprehensive or not described well. **0 points**. |
| 1. **Were the criteria used for deciding which studies to include in the review reported?** |
| 1. **Yes**, inclusion and exclusion criteria clearly defined. **2 points**. |
| 1. **Partially**, reference to inclusion and exclusion criteria can be found but are not defined clearly enough. **1 point**. |
| 1. **No**, no criteria defined. **0 points** |
| 1. **Was bias in the selection of articles avoided?** |
| 1. **Yes**, issues influencing selection bias were covered. Both of the following bias-avoiding strategies were used: (1) two or more assessors independently judged study relevance, (2) assessors selected studies using predetermined criteria. **2 points**. |
| 1. **Can’t tell**, only one of the strategies used. **1 point**. |
| 1. **No**, selection bias was not avoided or was not discussed. **0 points**. |
| 1. **Were the criteria used for assessing the methodological quality of studies reviewed reported?** |
| 1. **Yes**, criteria defined and used addressed the major factors influencing bias. **2 points**. |
| 1. **Partially**, some discussion or reference to criteria. **1 point**. |
| 1. **No**, validity or methodological quality criteria not used or not described. **0 points**. |
| 1. **Were study quality assessment criteria used to inform the review analysis?** |
| 1. **Yes**, criteria were used to inform the analysis, either by exclusion from the analysis if low quality or through sensitivity analysis. **2 points**. |
| 1. **Partially**, some discussion but not clearly described application of criteria. **1 point**. |
| 1. **No**, criteria not used or not described. **0 points**. |
| 1. **Were the methods used to combine the findings of the relevant studies (to reach a conclusion) reported?** |
| 1. **Yes**, qualitative and quantitative methods are acceptable. **2 points.** |
| 1. **Partially**, partial description of methods to combine and tabulate; not sufficient to duplicate. **1 point**. |
| 1. **No**, methods not stated or described. **0 points**. |
| 1. **Were findings of the relevant studies combined appropriately relative to the primary question of the overview?** |
| 1. **Yes**, combining of studies appears acceptable. **2 points**. |
| 1. **Can’t tell**, should be marked if in doubt. **1 point**. |
| 1. **No**, no attempt was made to combine findings, and no statement was made regarding the inappropriateness of combining findings. **0 points**. |
| 1. **Were the conclusions made by the author(s) supported by the data and/or analysis reported in the overview?** |
| 1. **Yes**, data were reported that support the main conclusions regarding the primary question(s) that the overview addresses. **2 points**. |
| 1. **Partially**. **1 point**. |
| (c) **No**, conclusions not supported or unclear. **0 points**. |

**Table S3: Evidence Grading System** (**Source: SIGN 50. A guideline developer’s handbook, 2008)**

| **LEVELS OF EVIDENCE QUALITY** |
| --- |
| 1++ High quality meta-analyses, systematic reviews of randomised control trials, or randomised control trials with a very low risk of bias |
| 1+ Well-conducted meta-analyses, systematic reviews, or randomised control trials with a low risk of bias |
| 1- Meta-analyses, systematic reviews, or randomised control trials with a high risk of bias |
| 2++ High quality systematic reviews of case control or cohort studies High quality case control or cohort studies with a very low risk of confounding or bias and a high probability that the relationship is causal |
| 2+ Well-conducted case control or cohort studies with a low risk of confounding or bias and a moderate probability that the relationship is causal |
| 2- Case control or cohort studies with a high risk of confounding or bias and a significant risk that the relationship is not causal |
| 3 Non-analytic studies, e.g. case reports, case series |
| 4 Expert opinion |

**CRITERIA FOR GRADING EVIDENCE QUALITY IN THIS REVIEW**

The evidence grading system applied to each analysis was based on the Scottish Intercollegiate Guidelines Network (SIGN) evidence grading system (as above), which is also used by the UK’s National Institute for Health and Clinical Excellence (NICE). However, we were looking at analyses which identified intervention characteristics associated with effectiveness, rather than effectiveness per se. We therefore refined the system as follows:-

1. Evidence Grade 1 (causal evidence) was assigned to randomised, between-group comparisons of individual-level data. Where reviews have assessed the quality of their component trials and taken this into account in analyses, this will tend to reduce the risks of bias in that analysis due to differences in population, intervention type, measurement and attrition. However, further risk may still arise from bias in the selection of trials to enter into the meta-analysis (e.g. poor categorisation of the component being analyzed) or other sources of possible error (e.g. low overall N). Based on these considerations, the overall risk of bias for each analysis was assessed by two reviewers (KS, CG) to assign levels of ‘++’, ‘+’ or ‘-’ to the evidence grade.
2. Evidence Grade 2 (associative evidence) was applied to observational comparisons or contrasts of study-level data (of types i to iv described below). The risks of bias due to study level factors (i.e. whether effects might be explained by differences in or covariance with study population, intervention type, study quality, measurement methods), as well as other factors (e.g. overall statistical power) was assessed by two reviewers (KS, CG) to assign levels of ‘++’, ‘+’ or ‘-’. Hence, Evidence Grade 2++ was applied to balanced observational comparisons where clear evidence was presented that the groups compared are well-matched such that there was a low risk of bias due to differences in population, intervention type, measurement and study quality, and there were no other likely sources of bias or unreliability. A grade of 2- was applied if there was clearly a high risk of bias for the particular comparison (e.g. comparison based on low numbers of participants/trials or there was clear bias in the selection of trials for the sub-group analysis, or clear imbalances between the groups compared).
3. An assignment of ‘ungraded’ was applied to any analysis (causal or associative) where there was a clear indication of serious methodological weakness (e.g. severe risk of bias), or if the total number of participants contributing to the analysis was less than 100.

APPLICATION OF THE GRADING SYSTEM IN THIS REVIEW:

The reviews which we selected used five different approaches to identify intervention characteristics associated with increased effect size:-

i) ‘Vote counting’. This involves dividing individual studies into groups according to the inclusion of particular characteristics (e.g. high vs. low intensity) and counting the number or proportions of studies which found statistically significant differences (i.e. the number of significant results for groups of trials with different intervention components were counted and compared numerically without statistical analysis).

ii) Stratified meta-analysis: Stratified meta-analysis was used to compare the effect sizes of trials which were grouped according to the inclusion of particular characteristics (e.g. high vs. low intensity) (i.e. the results for sub-groups of trials with different intervention components were statistically pooled and then compared numerically without further statistical analysis).

iii) Meta-regression: Meta-regression analysis was used to compare the effect sizes of trials which were grouped according to the inclusion of particular characteristics (e.g. high vs. low intensity) (i.e. the results for sub-groups of trials with different intervention components were statistically pooled and then statistically contrasted).

iv) Qualitative (descriptive) summaries: This typically involved looking at the intervention descriptions of successful and unsuccessful trials and qualitatively extracting themes which seem more common in the successful studies (e.g. the studies which found a significant difference were more likely to be intensive). This has some advantages in terms of the ability to identify more subtle patterns in the data, but also disadvantages in terms of not being able to produce any estimation of the likely size or statistical reliability of the effect implied.

v) Within study experimental comparison with randomisation: In these analyses, statistical comparisons had been made (in individual RCTs) between groups, which were randomised according to particular contrast characteristics (e.g. high intensity vs. low intensity). The results of such studies had then been summarised either descriptively, or, more usually, by meta-analysis.

The first four approaches are essentially observational, associative analyses (indirect post hoc observations of study-level results) and were therefore graded at level 2. The fifth approach derives from randomised, between group comparisons of the relevant factor and this type of analysis was graded at level 1.

## Table S4 - Characteristics of Included Reviews

| **Study** | **Type of review** | **Aim** | **Inclusion/exclusion criteria** | **Key outcomes** | **OQAQ Score**  **(see table S6)** | **Period searched** |
| --- | --- | --- | --- | --- | --- | --- |
| Ashworth et al. 2005 (30) | Descriptive | To assess the effectiveness of 'home-based' versus 'centre-based' physical activity interventions on the health of older adults | *Design*: Randomised or quasi-randomised controlled trials, comparing home-based and centre-based physical activity programmes  *Participants*: Adults (50yrs+) with cardiovascular risk factors | Physical activity | 17 | 1966 to Sept 2002 |
| Avenell et al. 2004 (31) | Meta-analysis | To review the long-term effects of obesity treatments (inc. diet & physical activity interventions) on body weight, risk factors for disease, and disease | *Design*: RCTs with detailed descriptions of an intervention programme; minimum 2 yr follow-up  *Participants*: Adults (18-70yrs) with BMI of 28 kg/m^2^ or more | Weight | 16 | 1966 to May 2001 |
| Bosch et al. 2007 (32) | Descriptive | To assess the effects of contracts between patients and healthcare practitioners on patients' adherence to treatment, prevention and promotion of healthy diet & physical activity | *Design*: RCTs  *Participants*: Patients or their carers, any age or gender, with any health condition in any health setting. Practitioners, and any worker or service providing screening, diagnosis, therapeutics, rehabilitation, prevention or health promotion activities | Weight | 17 | 1966 to May 2004 |
| Bravata et al. 2007 (33) | Meta-analysis | To evaluate the association of pedometer use with physical activity and health outcomes | *Design*: RCTs or observational studies, with more than 5 participants, reporting change in the number of steps / day.  *Participants*: Adult outpatients | Physical activity | 14 | 1966 to February 2007 |
| Brunner et al. 2007 (34) | Meta-analysis | To assess the effects of providing dietary advice to achieve sustained dietary changes or improved cardiovascular risk profile among healthy adults | *Design*: RCTs involving parallel group design  *Participants*: Healthy community dwelling adults (18yrs +) including 13 RCTs in people with cardiovascular risk factors | Dietary change | 15 | Jan 1966 to Nov 2006 |
| Burke et al. 2003 (35) | Meta-analysis | Evaluate the efficacy & sustained efficacy of adaptations of motivational interviewing (AMI) compared with control procedures and other active treatments | *Design*: Controlled clinical trials  *Participants*: Not reported | Physical activity & weight | 14 | nr |
| Curioni & Lourenco 2005 (36) | Meta-analysis | To assess the effectiveness of dietary interventions and exercise in long-term weight loss in overweight and obese people | *Design*: RCTs of diet, exercise or both, follow-up period after intervention of at least 1yr  *Participants*: Overweight and obese adults 18 years old or older with BMI of >25 | Weight | 14 | Inception to March 2003 |
| Dansinger et al. 2007 (37) | Meta-analysis | To perform a meta-analysis of the effect of dietary counselling compared with usual care on body mass index (BMI) over time in adults | *Design*: RCTs (≥16 weeks in duration) with min. observation period, including treatment & follow-up, of at least 1 year  *Participants*: Overweight or obese adults (18yrs+) | Weight | 17 | Jan 1997 to July 2006 |
| Dombrowski et al. 2008 (38) | Meta-analysis & descriptive | To identify intervention and programme features which are linked to more effective interventions in terms of behaviour (diet & physical activity) and weight change | *Design*: Published RCTs providing ≥12 wks follow-up data after the point of randomisation  *Participants*: Adults with BMI of ≥ 30, age of ≥ 40yr and one additional risk factor for morbidity | Physical activity, dietary change & weight loss | 15 | Studies published between 1985 and 2008 |
| Douketis et al. 2005 (39) | Descriptive | To investigate lifestyle (diet & physical activity), pharmacologic, and surgical methods of weight loss to assess (1) weight loss efficacy (2) effects of weight loss on cardiovascular risk factors  (3) applicability of findings from studies to everyday clinical practice | *Design*: RCTs or non-RCTs  *Participants*: Overweight or obese adults with BMI ≥25kg/m² | Weight | 15 | 1966 to September 2003 |
| Eakin et al. 2000 (40) | Meta-analysis & descriptive | To find out what strategies are practical and effective to use in primary care settings to enhance levels of patient physical activity | *Design*: RCTs or quasi-experimental study with a comparison group, intervention delivered or initiated in a primary care setting  *Participants*: Not reported | Physical activity | 14 | 1980 to 1998 |
| Foster et al. 2005 (41) | Meta-analysis | To assess the effects of interventions for promoting physical activity | *Design*: RCTs comparing different interventions to encourage sedentary adults not living in an institution to become physically active  *Participants*: Sedentary adults (16 yrs+), not living in an institution | Physical activity, cardio-respiratory fitness | 17 | January 1966 to December 2001 |
| Galani & Schneider 2007 (42) | Meta-analysis | To assess the mid- to long-term effectiveness of lifestyle interventions in the prevention and treatment of obesity | *Design*: RCTs with min. observation period, including treatment & follow-up, of at least 1 year  *Participants*: Overweight or obese adults (18yrs+) | Weight | 16 | 1995 to 2005 |
| Gillies et al. 2007 (43) | Meta-analysis | To quantify the effectiveness of pharmacological and lifestyle interventions to prevent or delay type 2 diabetes in people with IGT | *Design*: RCTs, studies had to have an intervention to delay or prevent type 2 diabetes  *Participants*: Individuals with IGT | Progression to type 2 diabetes | 17 | 1966 to July 2006 |
| Halcomb et al. 2007 (44) | Descriptive | To investigate the efficacy of general practice nurse interventions for cardiac risk factor reduction | *Design*: RCTs that investigated the effectiveness of interventions for cardiovascular disease management or risk factor reduction undertaken by general practice nurses  *Participants*: Adults (18yrs+) | Physical activity & weight | 14 | 1966 to 2005 |
| Kahn et al. 2002 (45) | Descriptive | To evaluate effectiveness of various interventions to increasing physical activity | *Design*: Intervention studies, RCTs or non-RCTs, multiple measurement before-and-after designs with concurrent comparison groups, prospective cohort studies  *Participants*: Not reported | Physical activity, aerobic capacity | 15 | 1980 to 2000 |
| McTigue et al. 2003 (46) | Descriptive | To assess the benefits and harms of screening and earlier treatment in reducing morbidity and mortality from overweight and obesity | *Design*: RCTs of good or fair quality of counselling and behavioural interventions, promoting change in diet or exercise or both  *Participants*: Overweight or obese (BMI ≥25) adults (18yrs+) | Weight | 16 | Jan 1994 to Feb 2003 |
| McTigue et al. 2006 (47) | Descriptive | To examine evidence concerning obesity's health-related risks, diagnostic methods, and treatment outcomes in older individuals | *Design*: RCTs with a follow-up of at least 1 year  *Participants*: Adults ≥ 60yrs | Weight | 16 | Jan 1st 1980 to Nov 2005 |
| Michie et al. 2008 (48) | Meta-analysis | To identify effective individual techniques and theoretically derived combinations of techniques which are linked to more effective interventions in terms of behaviour and weight change | *Design*: Experimental or quasi-experimental *Participants*: Adults (18yrs+) | Physical activity & dietary change | 15 | 1990 to 2007 |
| Murphy et al. 2007 (49) | Meta-analysis | To quantify changes due to walking interventions, that may alter cardiovascular risk factors | *Design*: RCTs with walking as the only intervention  *Participants*: Sedentary but apparently healthy adults (18yrs+) | Cardiovascular fitness & weight | 15 | 1971 to Sept 2004 |
| Norris et al. 2007 (50) | Meta-analysis | To assess the effectiveness of dietary, physical activity, and behavioural weight loss, and weight control intervention in adults with pre-diabetes | *Design*: RCTs with weight loss or weight control as their primary stated goal  *Participants*: Adults (18yrs +) with pre-diabetes, of any weight | Weight | 17 | 1966 to May 2004 |
| Ogilvie et al. 2007 (51) | Descriptive | To assess the effects of interventions to promote walking in individuals and populations. | *Design*: Any design  *Participants*: No limits imposed | Physical activity | 16 | 1990 onwards |
| Richardson et al. 2008 (52) | Meta-analysis | To examine the effects of pedometer-based walking interventions on weight loss | *Design*: RCTs or other controlled trials or pre-intervention and post-intervention prospective cohort study, studies using pedometers as motivational tool to increase walking  *Participants*: Sedentary, overweight or obese (>25kg/m²) adults | Weight | 15 | Search conducted July 2005 (period searched not reported) |
| Rubak et al. 2005 (53) | Meta-analysis | To evaluate the effectiveness of motivational interviewing (MI) in different areas of disease and to identify factors shaping outcomes | *Design*: RCTs using MI as the intervention  *Participants*: Not reported | Weight | 16 | 1963 to Jan 2004 |
| Shaw et al. 2005 (54) | Meta-analysis | To assess the effects of psychological interventions for overweight or obesity as a means of achieving sustained weight loss | *Design*: RCTs  *Participants*: Overweight or obese (BMI >25kg/m²) adults (18yrs+) | Weight | 17 | Inception to June 2003 |
| Shaw et al. 2006 (55) | Meta-analysis | To assess regular exercise as a means of achieving weight loss, using RCTs and focused on overweight and obese populations | *Design*: RCTs and quasi-RCTs only *Participants*: Adults (18yrs+) | Weight | 16 | Inception to 2003 |
| Thompson et al. 2003 (56) | Meta-analysis | To assess effects of dietary advice given by a dietician compared with another health professional, or use of self-help resources, in reducing blood cholesterol in adults | *Design*: RCTs of at least 6-wks intervention. All interventions had to include dietary advice to reduce blood cholesterol  *Participants*: Adults (18yrs+), participants with or without existing heart disease or previous MI | Weight | 18 | 1966 to Sept 2002 |
| Tsai & Wadden 2005 (57) | Descriptive | To describe the components, costs, and efficacy of the major commercial and organised self-help weight-loss programmes in the United States | *Design*: Any design conducted in the US  *Participants*: Adults | Weight | 14 | 1966 to 2003 |
| Whitlock et al. 2003 (58) | Descriptive review of reviews | To examine whether: (1) changing individual health behaviour improves health outcomes, and (2) interventions in the clinical setting influence people to change their behaviour | *Design*: RCTs or non-RCTs of primary care based interventions or primary care-feasible interventions conducted in clinical settings  *Participants*: Adult women | Physical activity & dietary change | 14 | 1996 to 2003 |
| Williams et al. 2007 (59) | Meta-analysis & descriptive | To assess whether exercise-referral schemes are effective in improving exercise participation in sedentary adults | *Design*: RCTs, non-RCTs, observational studies, process evaluations and qualitative studies  *Participants*: Adults referred to exercise-referral schemes from primary care | Physical activity | 17 | Inception to March 2007 |

**Table S5**: Excluded papers

| **Papers excluded** | **Reason(s) for exclusion*** |
| --- | --- |
| Obesity: weight loss without drugs: a balanced diet avoiding high- calorie foods, plus exercise. Prescrire International 2007; 16:162-167. | B |
| Adams J, White M. Are activity promotion interventions based on the trans-theoretical model effective? A critical review. Br J Sports Med 2003;37:106-114. | B |
| Allen NA. Social cognitive theory in diabetes exercise research: an integrative literature review. Diabetes Educator 2004;30:805-819 | D |
| Anderson J, Luan J, H°ie L. Structured weight-loss programs: meta-analysis of weight loss at 24 weeks and assessment of effects of intervention intensity. Advances in Therapy 2004;21(2):61-75. | B |
| Angelo JB, Huang J, Carden D. Diabetes prevention: a review of current literature. Adv Stud Med 2005; 5(5):250-259. | A |
| Astrup A, Grunwald GK, Melanson EL, Saris WH, Hill JO. The role of low-fat diets in body weight control: a meta-analysis of ad libitum dietary intervention studies. International Journal of Obesity and Related Metabolic Disorders: Journal of the International Association for the Study of Obesity 2000;24:1545-1552. | B |
| Ayyad C, Andersen T. Long-term efficacy of dietary treatment of obesity: a systematic review of studies published between 1931 and 1999. Obesity reviews 2000;1:113-119. | B |
| Bronner Y, Boyington J. Developing weight loss interventions for African-American women: elements of successful models. J Natl Med Assoc 2002;94:224-235. | B |
| Case J, Willoughby D, Haley Z, V, Maybee P. Today's educator. Preventing type 2 diabetes after gestational diabetes. Diabetes Educator 2006;32:877-878. | A |
| Catenacci V, Wyatt H. The role of physical activity in producing and maintaining weight loss. Nature clinical practice Endocrinology & metabolism 2007;3:518-529. | B |
| Curtis J, Wilson C. Preventing type 2 diabetes mellitus. J Am Board Fam Pract 2005;18:37-43. | B |
| Dachs R. Exercise is an effective intervention in overweight and obese patients. Am Fam Phys 2007;75:1333-1336. | A |
| Davies MJ, Tringham JR, Troughton J, Khunti KK. Prevention of Type 2 diabetes mellitus. A review of the evidence and its application in a UK setting. Diabetic Med 2004;21:403-414. | A |
| Eden K, Orleans T, Mulrow C, Pender N, Teutsch S. Does Counseling by Clinicians Improve Physical Activity? A Summary of the Evidence for the U.S. Preventive Services Task Force 2002; 137(3) 208-215 | A |
| Faith MS, Fontaine KR, Cheskin LJ, Allison DB. Behavioral approaches to the problems of obesity. Behav Modif 2000;24:459-493. | A |
| Fappa E, Yannakoulia M, Pitsavos C, Skoumas I, Valourdou S, Stefanadis C. Lifestyle intervention in the management of metabolic syndrome: could we improve adherence issues? Nutrition (Burbank Los Angeles County Calif ) 2008;24:286-291. | B |
| Fein SP, Sherman SE. Review: brief primary care interventions are moderately effective for increasing physical activity... commentary on Eakin EG, Glasgow RE, Riley KM. Review of primary care-based physical activity intervention studies. Effectiveness and implications for practice and future research. J Fam Pract 2000 Feb; 49:158-68. Evidence-based Nursing 4,45,2001. | A |
| Fogelholm M, Kukkonen H. Does physical activity prevent weight gain - a systematic review. Obesity Reviews 2000;1:95-111. | B |
| Fogelholm M, Lahti K. Community health-promotion interventions with physical activity: Does this approach prevent obesity? Scand J Nutr Naringsforsk 2002;46:173-177. | B |
| Foreyt JP, Poston WS. The role of the behavioral counselor in obesity treatment. Journal of the American Dietetic Association 1998; 98(Suppl. 2):S27-S30. | A |
| Franz M. Effectiveness of weight loss and maintenance interventions in women. Current Diabetes Reports 2004;4:387-393. | B |
| Franz M, Van Wormer J, Crain A, Boucher J, Histon T, Caplan W et al. Weight-loss outcomes: a systematic review and meta-analysis of weight-loss clinical trials with a minimum 1-year follow-up. Journal of the American Dietetic Association 2007;107:1755-1767. | B |
| Goetz P. Review: dietary advice improves dietary intake and reduces cardiovascular risk factors. Evidence-based Nursing 2006;9,48, doi:10.1136. | A |
| Goldstein M, Whitlock E, DePue J. Multiple behavioral risk factor interventions in primary care Summary of research evidence. Am J Prev Med 2004;27(2 Suppl):61-79. | B |
| Hamilton S, Hankey CR, Miller S, Boyle S, Melville CA. A review of weight loss interventions for adults with intellectual disabilities. Obesity reviews 2007;8:339-345. | B |
| Hardeman W, Griffin S, Johnston M, Kinmonth AL, Wareham NJ. Interventions to prevent weight gain: a systematic review of psychological models and behaviour change methods. International Journal of Obesity and Related Metabolic Disorders: Journal of the International Association for the Study of Obesity 2000;24:131-143. | D |
| Hillsdon M, Foster C, Cavill N, Crombie H, Naidoo B. The effectiveness of public health interventions for increasing physical activity among adults: a review of reviews London: Health Development Agency, 2005. | A |
| Horvath K, Jeitler K, Siering U, Stich AK, Skipka G, Gratzer TW et al. Long-term effects of weight-reducing interventions in hypertensive patients: systematic review and meta-analysis. Arch Int Med 2008;168:571-580. | F |
| Jepson R, Harris F, MacGillivray S, Kearney N, Rowa-Dewar N. A review of the effectiveness of interventions, approaches and models at individual, community and population level that are aimed at changing health outcomes through changing knowledge attitudes and behaviour. London: NICE; 2006. | A |
| Katz,D, Connell,M, Yeh,M, Nawaz,H, Njike,V, Anderson,L, Cory,S, Dietz,W. Public health strategies for preventing and controlling overweight and obesity in school and worksite settings: a report on recommendations of the Task Force on Community Preventive Services 2005. MMWR. Recommendations and reports: Morbidity and mortality weekly report. Recommendations and reports / Centers for Disease Control, 54 (RR-10):1-12. | B |
| Ketola E, Sipila R, Makela M. Effectiveness of individual lifestyle interventions in reducing cardiovascular disease and risk factors. Ann Med 2000;32:239-51. | F |
| King A, Rejeski W, Buchner D. Physical activity interventions targeting older adults: A critical review and recommendations. Am J Prev Med 1998;15:316-333. | B |
| Krummel DA, Koffman DM, Bronner Y, Davis J, Greenlund K, Tessaro I et al. Cardiovascular health interventions in women: What works? Journal of Women's Health & Gender-based Medicine 2001;10:117-136. | B |
| Lang A, Froelicher E. Management of overweight and obesity in adults: behavioral intervention for long-term weight loss and maintenance. European journal of cardiovascular nursing :Journal of the Working Group on Cardiovascular Nursing of the European Society of Cardiology 2006;5:102-114. | B |
| Lindberg N, Stevens V. Review: weight-loss interventions with Hispanic populations. Ethnicity & Disease 2007;17:397-402. | A |
| Lindner H, Menzies D, Kelly J, Taylor S, Shearer M. Coaching for behaviour change in chronic disease: A review of the literature and the implications for coaching as a self-management intervention. Aust J Prim Health 2003;9:177-185. | A |
| Melkus GD. Review: non-pharmacological interventions induce or maintain weight loss in adults with pre-diabetes. Evidence-based Nursing 2005;8:110,doi:10.1136. | A |
| Milner P, Hams SP, Markandya A, Shaw S, Ward Booth S Psychosocial interventions for the maintenance of weight loss in obese adults Cochrane Database Syst Rev: Protocols Issue 2 John Wiley & Sons, Ltd Chichester, UK, 2008. | A |
| Moore H, Summerbell CD, Hooper L, Ashton V, Kopelman P Dietary advice for the prevention of type 2 diabetes mellitus in adults Cochrane Database Syst Rev: Protocols Issue 1 John Wiley & Sons, Ltd Chichester, UK, 2005. | A |
| Morgan O. Approaches to increase physical activity: reviewing the evidence for exercise-referral schemes. Public Health 2005;119:361-370. | B |
| Orozco LJ , Mauricio D, Gimenez Perez G, Roque M Exercise or exercise and diet for preventing type 2 diabetes mellitus Cochrane Database Syst Rev: Protocols Issue 2 John Wiley & Sons , Ltd Chichester, UK, 2007. CD00305. | A |
| Pinto A, Gokee L, Wing R. Behavioral approaches to weight control: A review of current research. Womens Health 2007;3:341-353. | A |
| Pinto BM, Goldstein MG, Marcus BH. Activity counseling by primary care physicians. Prev Med 1998;27:506-513. | A |
| Pirozzo S, Summerbell C, Cameron C, Glasziou P. Advice on low-fat diets for obesity. Cochrane Database Syst Rev 2008;CD003640. | E |
| Pletcher MJ, Baron RB. Primary prevention of cardiovascular disease in women: new guidelines and emerging strategies. Adv Stud Med 2005;5:412-419. | A |
| Qvigstad E. Prevention of type 2 diabetes: An overview. Tidsskrift for den Norske Laegeforening 2004;124:3047-3050. | C |
| Saris WH. Very-low-calorie diets and sustained weight loss. Obesity Research 2001;9(Suppl 4):295S-301S. | A |
| Satterfield D, Volansky M, Caspersen C, Engelgau M, Bowman B, Gregg E et al. Community-based lifestyle interventions to prevent type 2 diabetes. Diabetes Care 2003;26:2643-2652. | B |
| Schroeder K, Fahey T, Ebrahim S. Interventions for improving adherence to treatment in patients with high blood pressure in ambulatory settings. Cochrane Database Syst Rev 2004;CD004804. | F |
| Seefeldt V, Malina R, Clark M. Factors affecting levels of physical activity in adults. Sports Med 2002;32:143-168. | A |
| Seo D, Sa J. A meta-analysis of psycho-behavioral obesity interventions among US multiethnic and minority adults. Prev Med 2008;(epub: 15 1 2008). | B |
| Sharma M. Behavioural interventions for preventing and treating obesity in adults. Obesity Reviews 2007;5:441-449. | B |
| Sharma A, Iacobellis G. Treatment of obesity: a challenging task. Contrib Nephrol 2006151:212-220. | B |
| Sherwood NE, Jeffery RW. The behavioral determinants of exercise: implications for physical activity interventions. Annu Rev Nutr 2000;20:21-44. | A |
| Slevin E. High intensity counselling or behavioural interventions can result in moderate weight loss. Evid Based Health 2004;8:136-138. | A |
| Sorensen JB, Skovgaard T, Puggaard L. Exercise on prescription in general practice: a systematic review. Scand J Prim Health Care 2006;24: 69-74. | B |
| Taylor AH, Cable NT, Faulkner G, Hillsdon M, Narici M, van d. Physical activity and older adults: a review of health benefits and the effectiveness of interventions. J Sports Sci 2004;22:703-725. | A |
| Taylor W, Baranowski T, Young D. Physical activity interventions in low-income, ethnic minority, and populations with disability. Am J Prev Med 1998;15:334-343. | B |
| Thorogood M. Combining diet with physical activity in the treatment of obesity... Proceedings from the ASO and BDA symposium held on 25 November 1997 at St. Bartholomew's Hospital, London. J Hum Nutr Diet 1998;11:239-242. | A |
| Touyz RM, Campbell N, Logan A, Gledhill N, Petrella R, Padwal R. The 2004 Canadian recommendations for the management of hypertension: Part III--Lifestyle modifications to prevent and control hypertension. Can J Cardiol 2004; 20:55-59. | A |
| Tufano J, Karras B. Mobile eHealth interventions for obesity: a timely opportunity to leverage convergence trends. Journal of Medical Internet Research 2005;7:e58. | A |
| United States Preventive Services Task Force: Behavioral Counseling in Primary Care to Promote a Healthy Diet: Recommendations and Rationale: United States Preventive Services Task Force. *The Internet Journal of Family Practice* 2002; 2. | A |
| Upchurch SL. Review: lifestyle or pharmacological interventions prevent or delay type 2 diabetes in people with impaired glucose tolerance. Evidence-based nursing 2007;10,78, doi:10.1136. | A |
| Verheijden MW, Bakx JC, van Weel C, Koelen MA, van Staveren WA. Role of social support in lifestyle-focused weight management interventions. Eur J Clin Nutr 2005;59(Suppl 1):S179-S186. | B |
| Viera A, Jamieson B. How effective are hypertension self-care interventions? J Fam Pract 2007;56:229-231. | B |
| Wadden T, Butryn M, Byrne K. Efficacy of lifestyle modification for long-term weight control. Obes Res 2004; 12:151S-162S. | A |
| Wadden T, Butryn M, Wilson C. Lifestyle modification for the management of obesity. Gastroenterology 2007;132:2226-2238. | B |
| Wadden T, Sarwer D. Behavioural treatment of the overweight patient. Baillieres Best Pract Clin Endocrinol Metab 1999;13:93-107. | A |
| Wareham N, van S, Ekelund U. Physical activity and obesity prevention: a review of the current evidence. Proc Nutr Soc 2005;64:229-247. | B |
| Weaver K. Review: little evidence supports the efficacy of major commercial and organised self help weight loss programmes. Evidence-based Nursing 2005;8,77, doi:10.1136. | A |
| Weinstein P. A review of weight loss programs delivered via the Internet. J Cardiovasc Nurs 2006;21:251-258. | B |
| Wilcox S, Parra M, Thompson R, Will J. Nutrition and physical activity interventions to reduce cardiovascular disease risk in health care settings: a quantitative review with a focus on women. Nutr Rev 2001;59:197-214. | B |
| Wing RR. Physical activity in the treatment of the adulthood overweight and obesity: current evidence and research issues. Med Sci Sports Exerc 1999;31(11 Suppl):S547-S552. | A |
| Wing RR, Tate DF. Lifestyle changes to reduce obesity. Curr Opin Endocrinol Diabetes 2000;7:240-246. | A |
| Yamaoka K, Tango T. Efficacy of lifestyle education to prevent type 2 diabetes. Diabetes Care 2005;28:2780-2786. | B |
| Yancey A, Kumanyika S, Ponce N, McCarthy W, Fielding J, Leslie J et al. Population-based interventions engaging communities of color in healthy eating and active living: a review. Preventing Chronic Disease 2004; 1:A09. | E |
| Yates T, Khunti K, Bull F, Gorely T, Davies MJ. The role of physical activity in the management of impaired glucose tolerance: a systematic review. Diabetologia 2007;50:1116-1126. | E |

**Key: A: Not a systematic review (36); B: OQAQ score <14 (33); C: Not in English (1); D: Inappropriate population (2) ; E: Review aims or intervention type not relevant (3); F: Inappropriate outcomes (2).**

**Table S6: OQAQ scores**

| **Study** | **OQAQ Score** | | | | | | | | | |
| --- | --- | --- | --- | --- | --- | --- | --- | --- | --- | --- |
|  | Qu 1 | Qu 2 | Qu 3 | Qu 4 | Qu 5 | Qu 6 | Qu 7 | Qu 8 | Qu 9 | **Total** |
| Ashworth et al. 2005 | 2 | 2 | 2 | 2 | 2 | 1 | 2 | 2 | 2 | 17 |
| Avenell et al. 2004 | 2 | 2 | 2 | 1 | 2 | 1 | 2 | 2 | 2 | 16 |
| Bosch et al. 2007 | 2 | 2 | 2 | 2 | 2 | 1 | 2 | 2 | 2 | 17 |
| Bravata et al. 2007 | 2 | 2 | 2 | 0 | 2 | 1 | 2 | 2 | 1 | 14 |
| Brunner et al. 2007 | 2 | 2 | 2 | 2 | 1 | 0 | 2 | 2 | 2 | 15 |
| Burke et al. 2003 | 1 | 2 | 2 | 1 | 1 | 2 | 2 | 1 | 2 | 14 |
| Curioni & Lourenco 2005 | 2 | 1 | 1 | 2 | 2 | 0 | 2 | 2 | 2 | 14 |
| Dansinger et al. 2007 | 2 | 1 | 2 | 2 | 2 | 2 | 2 | 2 | 2 | 17 |
| Dombrowski et al. 2008 | 2 | 1 | 2 | 2 | 2 | 0 | 2 | 2 | 2 | 15 |
| Douketis et al. 2005 | 2 | 2 | 2 | 2 | 2 | 1 | 1 | 1 | 2 | 15 |
| Eakin et al. 2000 | 2 | 2 | 2 | 1 | 1 | 1 | 2 | 1 | 2 | 14 |
| Foster et al. 2005 | 2 | 2 | 2 | 2 | 2 | 1 | 2 | 2 | 2 | 17 |
| Galani & Schneider 2007 | 2 | 2 | 2 | 0 | 2 | 2 | 2 | 2 | 2 | 16 |
| Gillies et al. 2007 | 2 | 2 | 1 | 2 | 2 | 2 | 2 | 2 | 2 | 17 |
| Halcomb et al. 2007 | 2 | 2 | 2 | 2 | 2 | 0 | 2 | 1 | 1 | 14 |
| Kahn et al. 2002 | 2 | 2 | 1 | 2 | 2 | 2 | 2 | 1 | 1 | 15 |
| McTigue et al. 2003 | 2 | 1 | 2 | 2 | 2 | 2 | 1 | 2 | 2 | 16 |
| McTigue et al. 2006 | 2 | 2 | 1 | 2 | 2 | 1 | 2 | 2 | 2 | 16 |
| Michie et al. 2008 | 2 | 2 | 2 | 2 | 1 | 0 | 2 | 2 | 2 | 15 |
| Murphy et al. 2007 | 2 | 2 | 2 | 1 | 1 | 1 | 2 | 2 | 2 | 15 |
| Norris et al. 2007 | 2 | 2 | 2 | 2 | 2 | 1 | 2 | 2 | 2 | 17 |
| Ogilvie et al. 2007 | 2 | 2 | 2 | 1 | 2 | 1 | 2 | 2 | 2 | 16 |
| Richardson et al. 2008 | 2 | 2 | 2 | 1 | 1 | 1 | 2 | 2 | 2 | 15 |
| Rubak et al. 2005 | 2 | 2 | 2 | 1 | 2 | 1 | 2 | 2 | 2 | 16 |
| Shaw et al. 2005 | 2 | 2 | 2 | 1 | 2 | 2 | 2 | 2 | 2 | 17 |
| Shaw et al. 2006 | 2 | 2 | 2 | 2 | 2 | 0 | 2 | 2 | 2 | 16 |
| Thompson et al. 2003 | 2 | 2 | 2 | 2 | 2 | 2 | 2 | 2 | 2 | 18 |
| Tsai & Wadden 2005 | 2 | 2 | 2 | 2 | 0 | 1 | 2 | 2 | 2 | 15 |
| Whitlock & Williams 2003 | 2 | 2 | 2 | 1 | 2 | 2 | 1 | 1 | 1 | 14 |
| Williams et al. 2007 | 2 | 1 | 2 | 2 | 2 | 2 | 2 | 2 | 2 | 17 |
