# Supplementary material for: Systematic review of reviews of intervention components associated with increased effectiveness in dietary and physical activity interventions
Source: BMC Public Health. 2011 Feb 18;11:119. doi: 10.1186/1471-2458-11-119 (PMC3048531; doi:10.1186/1471-2458-11-119)
Supplement: Additional file 2 — Tables S7-14: Data from analyses of: S7) Intervention Effectiveness; S8) Theoretical basis; S9) Behaviour change techniques; S10) Mode of delivery; S11) Intervention provider; S12) Intervention intensity; S13) Intervention population; S14) Intervention setting. [file 1471-2458-11-119-S2.DOC]

**Additional file 2: Evidence tables**

**Supplementary Tables S7-14**: Data from analyses of: S7) Intervention effectiveness; S8) Theoretical basis; S9) Behaviour change techniques; S10) Mode of delivery; S11) Intervention provider; S12) Intervention intensity; S13) Intervention population; S14) Intervention setting

| **Table S7: Intervention Effectiveness** | | | | | | | | |
| --- | --- | --- | --- | --- | --- | --- | --- | --- |
| **Study** | **Comparisons** | **Method of comparison** | **N studies (N participants)** | **Outcome** | **Follow-up time** | **Results** | **Evidence Grade** | **OQAQ Review Quality**  **(out of 18)** |
| **1. Changes in weight or BMI** | | | | | | | | |
| Norris et al. 2007 | Dietary and /or physical activity interventions versus control | Meta-analysis of RCTs making this comparison | 4 (1016) | Weight (kg) & BMI (kg/m^2^) | 1 yr | Interventions reduced weight by 2.8 kg (95% CI 1.0 to 4.7) (3.3% of baseline weight) and decreased BMI by 1.3 kg/m^2^ (95% CI, 0.8 to 1.9) | 1++ | 17 |
| Norris et al. 2007 | Dietary and /or physical activity interventions versus control | Meta-analysis of RCTs making this comparison | 3 (700) | Weight (kg) | 2 yrs | Interventions reduced weight by 2.6 kg (95% CI: 1.9 to 3.3) | 1++ | 17 |
| Galani & Schneider 2007 | Dietary and physical activity intervention versus standard care | Meta-analysis of RCTs making this comparison | 13 (3566) | Weight (kg) | 1 yr to 7 yrs (median 2 yrs) | Interventions reduced weight by a net –2.19 kg (95%CI: -2.81 to -1.57, p<0.0001) | 1++ | 16 |
| Douketis et al. 2005 | Dietary and /or physical activity interventions versus control | Summary of RCT & non-RCT mean differences using descriptive statistics (not meta-analysis) | 1) 12 (6432)  2) 6 (1743) | Weight (kg) | 1) 2 to 3 yrs (median not available)  2) 4 to 7 yrs (median not available) | 1) Intervention resulted in a mean net weight loss (±SD) of -3.5±2.4 kg based on a completers method of analysis  2) Intervention resulted in a mean net weight loss (±SD) of -3.6±2.6 kg based on a completers method of analysis | 1) 1+  2) 1+ | 15 |
| Avenell et al. 2004 | Diet, exercise and behaviour therapy compared to control | Meta-analysis of RCTs making this comparison | 1) 11 (1956)  2) 4 (1928)  3) 4 (1440)  4) 2 (164)  5) 1 (1101) | Weight (kg) | 1) 12mths  2) 18mths  3) 24mths  4) 30mths  5) 36mths | Intervention produced net weight changes (with 95% CIs) of:  1) -4.00 kg (-4.46 to -3.54)  2) -3.40 kg (-3.84 to -2.97)  3) -3.00 kg (-3.59 to -2.40)  4) -4.68 kg (-6.08 to -3.28)  5) -2.00 kg (-2.66 to -1.34) | 1,2&3) 1+    4&5) 1- | 16 |
| Dombrowski et al. 2008 | Behavioural intervention versus usual care | Meta- analysis of RCTs making this comparison | 23 (5020) | Weight (kg) | End of ‘active phase’ of intervention (Range 1 to14, mean 6.2 mths) | Intervention resulted in net changes of -3.0 kg (95%CI: -4.3 to -1.8) | 1++ | 15 |
| Dombrowski et al. 2008 | Behavioural intervention versus usual care | Meta- analysis of RCTs making this comparison | 11 (3689) | Weight (kg) | End of intervention maintenance phase (Range 6 to 36, mean 18.9 mths) | Intervention resulted in net changes of -3.6 kg (95%CI: -5.3 to -1.9) | 1++ | 15 |
| Dombrowski et al. 2008 | Behavioural intervention versus usual care | Meta- analysis of RCTs making this comparison | 7 (749) | Weight (kg) | No-intervention follow-up phase (Range: 12 to 24, mean: 16.7 mths) | Intervention resulted in net changes of -1.3 kg (95%CI: -2.6 to 0.1) | 1++ | 15 |
| Murphy et al. 2007 | Walking intervention versus control | Meta-analysis of RCTs making this comparison | 18 (792) | Weight (kg) | 8 to 52 wks, (median 24 wks) | Significant net weight loss of -0.95 kg (SD=0.61 kg, p<0.001) was observed after the walking programs. This represents a relative reduction of 1.4% in body weight | 1+ | 15 |
| Murphy et al. 2007 | Walking intervention versus control | Meta-analysis of RCTs making this comparison | 16 (816) | BMI (kg/m^2^) | 8 to 52 wks, (median 24 wks) | Significant net change in BMI of -0.28 kg/m^2^ (SD 0.2 kg/m^2^, p<0.05) was observed after the walking programs. This represents a relative reduction of 1.1% in BMI | 1+ | 15 |
| Shaw et al. 2006 | Exercise intervention versus no treatment control | Meta-analysis of RCTs making this comparison | 2 (270) | Weight (kg) | 12 mths | Intervention produced a net weight change of -2.03 kg (95%CI: -2.82 to -1.23) | 1- | 16 |
| Shaw et al. 2006 | Exercise intervention versus no treatment control | Meta-analysis of RCTs making this comparison | 2 (170) | BMI (kg/m^2^) | 6 mths and 12 mths | Intervention produced a net BMI change of -0.73 kg/m^2^ (95%CI: -0.99 to -0.46) | 1- | 16 |
| Williams et al. 2007 | Exercise referral scheme versus control | Descriptive summary of individual RCT results | 3 (1351) | BMI (kg/m^2^) | 37wks to 1yr (median 1yr) | No study reported any significant differences in BMI between exercise and control groups (no data provided) | 1+ | 17 |
| Dansinger et al. 2007 | Dietary counseling intervention versus control | Meta-analysis of RCTs making this comparison | 27 (1363) | BMI (kg/m^2^) | 1 yr | Mean net reduction in BMI of -1.88 kg/m^2^ (95% CI: -2.29 to -1.49) equating to 6% of initial body weight [-5.1 kg] | 1++ | 17 |
| Dansinger et al. 2007 | Dietary counseling intervention versus control | Meta-analysis of RCTs making this comparison | 4 (1807) | BMI (kg/m^2^) | 3yrs | Mean net reduction in BMI of -1.07 kg/m^2^ (95%CI: -1.67 to -0.47), equating to 3.4% of initial body weight (-2.9 kg] | 1++ | 17 |
| Tsai & Wadden 2005 | Weight Watchers program versus self-help intervention | Descriptive (single RCT) | 1 (423)  1 (309) | Weight (%) | 1) 1yr  2) 2yrs | 1) Participants lost 5.3% of their initial weight compared with 1.5% in the self-help arm (*p* < 0.001)  2) A weight loss of 3.2% was maintained at 2 yrs, compared with 0.0% in the self-help arm (*p* < 0.001) based on analysis of completers only | 1) 1-  2) 1- | 15 |
| Tsai & Wadden 2005 | Medically supervised proprietary diet program. Within-participant changes pre and post intervention | Descriptive summary of individual study results (1 RCT, 4 observational studies) | 1) 5 (1048)  2) 2 (557)  3) 1 (85)  4) 1 (100)  5) 1 (306) | Weight (%) | 1) 3 to 6 mths  2) 1 yr  3) 2 yrs  4) 3 yrs  5) 4 yrs | Interventions providing a low-calorie or very-low-calorie diet produced weight loss of approximately:-  1) 15% to 27% of initial weight  2) 8 to 9%  3) 15%  4) 7%  5) 5% | 1) 2-  2) 2-  3) Ungraded  4) 2-  5) 2 | 15 |
| **2. Changes in physical activity** | | | | | | | | |
| Foster et al. 2005 | Physical activity intervention versus control  1) continuous measures  2) dichotomous measures | Meta-analysis of RCTs making this comparison | 1) 19 (7598)  2) 10 (3595) | 1) Self-reported physical activity  2) Self-reported achievement of physical activity targets | min. 6 mths (median not available) | 1) The pooled effect size was moderate^a^ (SMD 0.28, 95% CI 0.15 to 0.41)  2) The pooled odds ratio for achieving target levels of physical activity was 1.33 (95% CI 1.03 to 1.72) | 1) 1++  2) 1++ | 17 |
| Foster et al. 2005 | Physical activity intervention versus control | Meta-analysis of RCTs making this comparison | 11 (2195) | Objectively measured cardio-respiratory fitness | min. 6 mths  (median not available) | The pooled effect size was moderate-to-strong (SMD 0.52, 95% CI 0.14 to 0.90) | 1++ | 17 |
| Dombrowski et al. 2008 | Behavioural intervention versus usual care | Meta- analysis of RCTs making this comparison | 21 (3048) | Standardised mean difference (Hedge’s adjusted g) for  objective or self-report measures of physical activity | End of ‘active phase’ of intervention (Range 1 to14 mths; mean 6.2 mths) | Intervention produced a moderate effect size (SMD 0.4, 95% CI 0.3 to 0.5) | 1+ | 15 |
| Dombrowski et al. 2008 | Behavioural intervention versus usual care | Meta- analysis of RCTs making this comparison | 9 (1444) | Standardised mean difference (Hedge’s adjusted g) for  objective or self-report measures of physical activity | End of intervention maintenance phase (Range 6 to 36 mths; mean 18.9 mths) | Intervention produced a moderate effect size (SMD = 0.3, 95% CI 0.1 to 0.5) | 1+ | 15 |
| Dombrowski et al. 2008 | Behavioural intervention versus usual care | Meta- analysis of RCTs making this comparison | 3 (458) | Standardised mean difference (Hedge’s adjusted g) for  objective or self-report measures of physical activity | No-intervention follow-up phase (Range: 12 to 24 mths, mean: 16.7 mths) | Intervention produced moderate effect size (SMD = 0.3, 95% CI 0.1 to 0.5) | 1+ | 15 |
| Ogilvie et al. 2007 | Walking intervention versus control (and observational study data) | Descriptive summary of individual study results | 27 (8764) with 17 RCTs | Self-reported or pedometer-recorded walking (minutes/week) | 6 wks to 10 yrs (median 6mths) | 15 of 27 studies (12 of 17 RCTs) found significant increases in physical activity. Typical increases for successful RCTs were 30-60 mins per week of additional physical activity, compared with controls | 1- | 16 |
| Williams et al. 2007 | Exercise referral scheme versus control | Meta-analysis of RCTs making this comparison | 5 (1923) | Physical activity  (nr^b^) | 10 wks to 24 mths (median 52 wks) | The relative risk of achieving 90 to 150 mins/wk moderate-intensity activity was 1.20 (95%CI: 1.06 to 1.35) in favour of exercise referral on an intention-to-treat basis. Number needed to treat = 17.2 | 1++ | 17 |
| Eakin et al. 2000 | Primary care-based physical activity interventions versus control | Descriptive summary of individual RCT & quasi-experimental study results | 10 (4170) | Self-reported physical activity (standardised effect size for continuous outcomes, odds ratio for categorical outcomes) | Up to 12 mths (median 6 wks) | 7 out of 10 studies reported statistically significant short-term outcomes. Effect sizes were small^a^ (SMD= 0.00 to 0.26; typical value of 0.26 for successful studies) and odds ratio from 1.04 to 3.73 (median 1.88, typically OR=1.48 for ‘now active rather than sedentary’ | 1- | 14 |
| Eakin et al. 2000 | Primary care-based physical activity interventions versus control | Descriptive summary of individual RCT & quasi-experimental study results | 7 (23,573) | Self-reported physical activity (standardised effect size for continuous outcomes, odds ratio for categorical outcomes) | >=12 mths (median 12 mths) | 3 out of 7 studies reported statistically significant longer-term outcomes. Effect sizes were typically small^a^ (SMD = 0.09) for successful studies). Odds ratio from 0.92 to 1.39 (median 1.25, typically OR=1.28 for ‘now active rather than sedentary’. No examples of successful intervention beyond 12 months | 1- | 14 |
| **3. Changes in dietary intake** | | | | | | | | |
| Brunner et al. 2007 | Dietary intervention versus control | Meta-analysis of RCTs making this comparison | 1) 18 (6170)  2) 15 (8416)  3) 7 (2981) | Self-reported dietary intake  1) fat  2) fruit & vegetables  3) dietary fibre | 3mths to 4 yrs  (median 12mths) | Interventions produced net changes in  1) Fat intake of -4.49% (95%CI: -2.31 to -6.66)  2) Fruit & vegetable consumption of +1.25 servings (95%CI: 0.70 to 1.81)  3) Dietary fibre of+5.99 g/day (95%CI: 1.12 to 10.86) | 1) 1-  2) 1-  3) 1- | 15 |
| Halcomb et al. 2007 | Practice nurse advice versus usual care | Descriptive summary of individual RCT results | 5, of which 2 with CVD patients (2580) | Self-reported dietary intake | 4mths to 4yrs (median 12mths) | 4 out of 5 studies (2 of 3 non-CVD studies) showed significant net changes in dietary intake (e.g. 5.7% change in absolute fat intake at 4 mths, 5% change in saturated fat intake at 1 & 3 yrs for the 2 non-CVD studies). Only 1 of 4 studies reported a significant effect at 12 mths or more (and only 1 of the 3 non-CVD studies) | Ungraded | 14 |
| Dombrowski et al. 2008 | Behavioural intervention versus usual care | Meta- analysis of RCTs making this comparison | 1) 13 (1686)  2) 16 (2468) | 1) Self-reported total energy consumption (kcal)  2) Self-reported fat consumption (Standardised mean difference) | End of ‘active phase’ of intervention (Range 1 to14 mths; mean 6.2 mths) | Intervention resulted in net changes of:  1) -112kcal ( 95% CI: -217 to -7)  2) SMD = -0.5 (95% CI: -0.7 to -0.2) | 1) 1+  2) 1+ | 15 |
| Dombrowski et al. 2008 | Behavioural intervention versus usual care | Meta- analysis of RCTs making this comparison | 1) 6 (1117)  2) 7 (2962) | 1) Self-reported total energy consumption (kcal)  2) Self-reported fat consumption (Standardised mean difference) | End of intervention maintenance phase (Range 6 to 36 mths; mean 18.9 mths) | Intervention resulted in net changes of:  1) -118 kcal (95% CI -178 to 57)  2) SMD = -0.4 (95% CI -0.7 to -0.2) | 1) 1+  2) 1+ | 15 |
| Dombrowski et al. 2008 | Behavioural intervention versus usual care | Meta- analysis of RCTs making this comparison | 1) 2 (432)  2) 3 (474) | 1) Self-reported total energy consumption (kcal)  2) Self-reported fat consumption (Standardised mean difference) | No-intervention follow-up phase (Range: 12 to 24 mths, mean: 16.7 mths) | Intervention resulted in net changes of:  1) -75 kcal (95% CI -189 to 40)  2) SMD = -0.2 (95% CI -0.7 to 0.3) | 1) 1+  2) 1+ | 15 |
| **4. Other outcomes** | | | | | | | | |
| Michie et al. 2008 | 1) Physical activity (PA) and healthy diet (HD) intervention versus control  2) separate effects of PA and HD interventions | Meta- analysis of RCTs making this comparison | 1) 71 (28,838)  2) PA: 44 (nr)  HD: 40 (nr) | Standardised mean difference (Cohen’s d) for objective or self-report measures of diet and physical activity (outcomes were combined as SMDs) | 1 wk to 24 mths (mean 6 mths) | 1) A moderate, significant effect was found, favoring the intervention (SMD = 0.37, 95% CI: 0.29, 0.45), but with substantial heterogeneity (I^2^ = 79%) 2) Individually, the review found that PA and HD interventions have moderate effect sizes; SMD = 0.34 (95% CI: 0.26, 0.43) and 0.38 (95% CI: 0.25, 0.52), respectively | 1) 1-  2) 1- | 15 |
| Gillies et al. 2007 | Lifestyle (diet and/or physical activity) intervention versus control | Meta- analysis of RCTs making this comparison  1) All RCTs  2) Diet-only  3) Exercise-only  4) Diet-and-exercise | 1) 10 (5885)  2) 3 (133)  3) 2 (193)  4) 7 (1592) | Development of type 2 diabetes | 1) 1.8 to 4.6 yr (mean 3.4)  2) Median 4.3 yrs  3) Median 3.6 yrs  4) Median 3.2 yrs | 1) Interventions produced a 49% relative reduction in risk of developing diabetes (hazard ratio 0.51; 95% CI: 0.44 to 0.60). Difference in absolute diabetes incidence -15.8% (95% CI: -19.8 to -11.9). Number needed to treat 6.4 (95%CI: 5.0 to 8.4)  2) Diet-only: hazard ratio = 0.67 (95%CI: 0.49, to 0.92)  3) Exercise: hazard ratio = 0.49 (95%CI: 0.32 to 0.74)  4) Diet-and-exercise: hazard ratio = 0.49 (95%CI: 0.40 to 0.59) | 1) 1++  2) 1++  3) 1++  4) 1++ | 17 |
| Dansinger et al. 2007 | Effectiveness of diet and/or physical activity intervention versus control in  1) Active phase  2) Maintenance | Stratified meta-analysis of RCTs | 1) 29 (nr. Estimate 7470)  2) 17 (nr. Estimate 4380) | Slope of net BMI change during active and maintenance phases | 1) 3 to 36 mths (median 12)  2) 6 to 60 mths (median 18) | 1) Active phase: weight loss from 3-12 mths was statistically significant at 0.08 BMI unit/month (p < 0.01)    2) Maintenance phase: weight regain from 6- 60 mths, of 0.03 BMI unit/mth (p<0.001) | 1++ | 17 |

Abbreviations: RCT = Randomised Controlled Trial. SMD = Standardised Mean Difference. BMI = Body Mass Index (kg/m2). SD = Standard Deviation. OQAQ = Oxman Quality Assessment Questionnaire, MTHS = Months, WKS = Weeks, NR = not reported, PA = Physical activity, HD = Healthy diet

^a^ Where reported, standardised effect sizes are categorised as small (SMD =0 up to 0.2); moderate (SMD = 0.2 up to 0.5); strong (SMD = 0.5 and above).

^b^ Type of physical activity measure analysed (self-report or other) not specified.

| **Table S8: Theoretical Basis** | | | | | | | | |
| --- | --- | --- | --- | --- | --- | --- | --- | --- |
| **Study** | **Comparisons** | **Method of comparison** | **N studies (N participants)** | **Outcome** | **Follow-up time** | **Results** | **Evidence Grade** | **OQAQ Review Quality**  **(out of 18)** |
| Dombrowski et al. 2008 | Theory-based intervention versus intervention with no stated theory base | Uni-variate meta-regression | 44 (10,560) | Weight (kg) | Active intervention phase (mean 6.2 mths) | Those studies which stated a theoretical model as the foundation of the intervention showed no trend in inducing greater weight losses compared to studies that did not state theoretical underpinnings | 2+ | 15 |
| Dombrowski et al. 2008 | Interventions congruent with different theoretical bases | A series of uni-variate meta-regression analyses (studies grouped according to no. of theory congruent techniques used) | 44 (10,560) | Weight (kg) | Active intervention phase (mean 6.2 mths) | Only Control Theory showed an increase in weight loss with the inclusion of more theory congruent techniques. This was marginally significant between studies using 3 Control Theory techniques (WMD = -4.7kg, 95%CI: -7.0 to -2.4) and those using none (WMD = -2.9kg, 95%CI: -4.6 to -1.2). All other theories showed no significance or marginal trends across studies | 2+ | 15 |
| Michie et al. 2008 | Interventions using behaviour change techniques congruent with Control Theory^28^ versus other interventions | Multi-variate meta- regression of RCT data | 71 (28,838) with 28 ‘congruent’ interventions | Standardised mean difference (Cohen’s d) for combined dietary and physical activity outcomes | 1 wk to 24 mths (mean 6 mths) | Results showed that interventions which prompted self-monitoring and used at least one other technique congruent with Control Theory generated around twice the effect size of other interventions (SMD = 0.60 (95%CI: 0.39 to 0.81) & 0.26 (95%CI: 0.20 to 0.31), respectively). Similar analyses restricted to only dietary and PA interventions found a similar pattern of results (SMD = 0.72 vs 0.24 and SMD = 0.50 vs 0.28 respectively). Sensitivity analyses excluding outliers and controlling for the number of non Control Theory related techniques suggested that the results were robust | 2+  2+  2+ | 15 |

Abbreviations: RCT = Randomised Controlled Trial. SMD = Standardised Mean Difference. WMD = Weighted Mean Difference. OQAQ = Oxman Quality Assessment Questionnaire, Mths = months

| **Table S9: Behaviour Change Techniques** | | | | | | | | |
| --- | --- | --- | --- | --- | --- | --- | --- | --- |
| **Study** | **Comparisons** | **Method of comparison** | **N studies (N participants)** | **Outcome** | **Follow-up time** | **Results** | **Evidence Grade** | **OQAQ Review Quality**  **(out of 18)** |
| **1. Use of established behaviour change techniques (non specific)** | | | | | | | | |
| Shaw et al. 2005 | Adding behaviour therapy to diet & exercise | Descriptive summary and meta-analysis of RCTs making this comparison | 6 (467) | Weight (kg) | 6 to 16 mths  (median 6mths) | 5 out of 6 studies (N=431) favoured adding behaviour therapy to diet & exercise, one (N=36) favoured diet and exercise alone. Meta-analysis found a benefit of adding behaviour therapy of -4.46kg (95%CI: -4.57 to -4.34) with significant heterogeneity between studies | 1+ | 17 |
| McTigue et al. 2003 | Adding behaviour therapy to diet & exercise | Descriptive summary of groups of RCTs and cohort studies with different intervention content | 14 (7776) | Weight (kg) | 12 to 54 mths  (median 12 mths) | Having a behavioural component in the intervention was associated with increased effectiveness, but this was almost perfectly confounded with increased contact frequency. The mean net weight change for the 11 (N=6097) behavioural (and higher intensity) interventions ranged from -3 to -5 kg, compared with an overall net weight change of -2.0 kg  Focusing on RCTs, of the 6 with a behavioural component, 4 achieved -2.5 to -5.5 kg net weight change (N=5482) and 2 achieved only borderline weight reduction (N=184). For non-behavioural interventions, net weight change ranged from -0.1 to -0.9 kg (3 studies, N=1759) | 2- | 16 |
| McTigue et al. 2006 | Adding behaviour therapy to diet and /or exercise for older people | Descriptive ‘vote-counting’ of significant RCT results | 11 (nr) | Weight (kg) | 12 to 48 mths  (median 15 mths) | Of 7 RCTs that included a behavioural component, 5 showed a significant or borderline significant weight loss. The four studies without a clear behavioural component showed no significant treatment effect on weight loss | 2+ | 16 |
| Avenell et al. 2004 | Adding behaviour therapy to diet | Meta-analysis of RCTs making this comparison | 1) 2 (50)  2) 1 (31)  3) 1 (34)  4) 1 (40) | Weight (kg) | 1) 12mths  2) 18ths  3) 36mths  4) 60mths | Adding behaviour therapy to diet improved weight loss (95%CI) by:-  1) -7.67 kg (-11.97 to -3.36)  2) -4.18 kg (-8.32 to -0.04)  3) -2.91 kg (-8.60 to 2.78)  4) 1.90 kg (-3.75 to 7.55) | Ungraded | 16 |
| Avenell et al. 2004 | Adding behaviour therapy to diet & exercise | Meta-analysis of RCTs making this comparison | 1 (105) | Weight (kg) | 12mths | The addition of behaviour therapy to diet & exercise did not significantly improve weight loss. NB: There were many groups in this study with N = ~13 per group, so this finding is not robust | Ungraded | 16 |
| Shaw et al. 2005 | Adding cognitive behavioural therapy to diet & exercise | Meta-analysis of RCTs making this comparison | 2 (63) | Weight (kg) | 4.5 and 6 mths | Adding CBT to exercise & diet resulted in significantly more weight loss (-4.9 kg, 95% CI: -7.3 to -2.4) | Ungraded | 17 |
| Michie et al. 2008 | Number of behaviour change techniques (BCTs) included in intervention | Uni-variate & multi-variate regression of RCT data | 71 (28,838) | Standardised effect size for dietary and physical activity outcomes (mix of objective and self-reported) | 1 wk to 24 mths (mean 6 mths) | The number of BCTs had no significant association with effect size (p > 0.05) | 2+ | 15 |
| Dombrowski et al. 2008 | Number of BCTs included in *dietary* interventions  4 sub-groups  (1-3, 4-6, 7-9, ≥10) | Stratified meta-analysis and between group comparisons using uni-variate meta-regression of RCT data | A) 4(394)  6(904)  8(802)  5(2920)  B) 1(54)  1(25)  2(81)  7(3529) | Weight (kg) | A) active intervention (1 to 14 mths, mean 6 mths)  B) maintenance phase (6 to 36 mths, mean 19mths) | A) In dietary interventions, more BCTs were associated with more weight loss, with 1-3 BCTs (-1.1 kg, 95% CI: -2.1 to 0); 4-6 BCTs (-1.8 kg, 95% CI: -3.2 to -0.5); 7-9 BCTs (-5.0 kg, 95% CI: -7.4 to -2.6)) and 10+ BCTs (-3.0 kg, 95% CI: -5.2 to 0.9). Sub group comparisons approached significance for using 1-3 BCTs compared with 7-9 BCTs (p = 0.052)  B) In the maintenance phase, weight loss was highest in studies which used 7-9 BCTs to change dietary behaviour. 1-3 BCTs (-3.3 kg, 95% CI: -5.7 to -1.0)); 4-6 BCTs (-2.6 kg, 95% CI: -7.5 to 2.3)); 7-9 BCTs (-7.9 kg, 95% CI: -11.1 to -4.7)) and 10+ BCTs (-2.9 kg, 95% CI: -4.9 to -0.8) | A) 2+  B) 2- | 15 |
| Dombrowski et al. 2008 | Number of BCTs included in *physical activity* interventions  4 sub-groups  (1-3, 4-6, 7-9, ≥10). | Stratified meta-analysis and between group comparisons using meta-regression techniques | A) 4(394); 6(904); 8(802); 5(2920)  B) 0 (0); 4(607); 1(59); 6(3023) | Weight (kg) | A) active intervention (1 to 14 mths, mean 6 mths)  B) maintenance phase (6 to 36 mths, mean 19mths) | A) In physical activity interventions, the highest weight loss was observed for studies that used 1-3 BCTs with 1-3 BCTs (-3.9 kg, 95% CI: -7.2 to -0.5); 4-6 BCTs (-1.8 kg, 95% CI: -2.9 to -0.7); 7-9 BCTs (-2.7 kg, 95% CI: -5.4 to 0.1)) and 10+ BCTs (-3.4 kg, 95% CI: -5.1 to -1.6). Subgroup comparisons were not significant (p > 0.3)  B) In the maintenance phase, studies using 7-9 BCTs showed the greatest weight loss. 1-3 BCTs (n/a); 4-6 BCTs (-3.5 kg, 95% CI: -5.4 to -1.6); 7-9 BCTs (-7.1 kg, 95% CI: -10.9 to -3.3) and 10+ BCTs (-2.9 kg, 95% CI: -5.4 to -0.4) | A) 2+  B) 2- | 15 |
| **2. Use of specific behaviour change techniques** | | | | | | | | |
| Avenell et al. 2004 | Social support (usually from family) versus same intervention individually delivered | Meta-analysis of RCTs making this comparison | 1) 4 (127)  2) 2 (209)  3) 1 (27)  4) 1 (20)  5) 1 (19) | Weight (kg) | 1) 12mths  2) 18mths  3) 24mths  4) 43mths  5) 48mths | Adding social /family support to interventions improved weight loss (95%CI) by:-  1) -2.96 kg (-5.31 to -0.60)  2) -1.80 kg (-3.04 to 0.87)  3) -5.61 kg (-10.98 to -0.24)  4) -0.75 kg (-6.95 to 5.45)  5) -1.55 kg (-7.88 to 4.78) | 1) 1+  2) 1-  3, 4 & 5) Ungraded | 16 |
| Michie et al. 2008 | Use versus non-use of specific behaviour change techniques (BCTs) | Multi-variate & uni-variate meta-regression of RCT data | 71 (28,838) | Standardised effect size for dietary and physical activity outcomes (mix of objective and self-reported) | 1 wk to 24 mths (mean 6 mths) | In the uni-variate analysis, only one of 26 techniques, namely ‘*prompt self-monitoring of behaviour’* was significantly associated with effect size (p < 0.05), explaining 14.6% of between study heterogeneity. The multi-variate model showed this association to be independent of setting, intensity, No. of BCTs, duration, population and delivery mode | 2+ | 15 |
| Dombrowski et al. 2008 | Use versus non-use of specific behaviour change techniques (BCTs) aimed at changing dietary behaviour | Stratified meta-analysis and uni-variate meta-regression of RCT data | N varies by BCT; 1) 1 to 22, typically 5-10 with BCT included (N=144 to 4523)  2) 1 to 10, typically 2-5 studies with BCT included (N=25 to 3610) | Weight (kg) | 1) active intervention: 1 to 14 mths, (mean 6 mths)  2) maintenance phase: 6 to 36 mths (mean 19mths) | 1) Three BCTs were significantly associated with net weight loss: T8 (provide instruction, -2.8 kg), T12 (prompt self-monitoring of behaviour, -3.4 kg), and T23 (relapse prevention, -2.8 kg) explaining 26.6%, 31.1% and 19.6% of the between-study heterogeneity respectively    2) In the maintenance phase only 1 BCT was significantly associated with net weight loss (T22 prompt self talk, -3.4 kg) although only 1 study was identified as having used this BCT | 1) 2+  2) 2+ | 15 |
| Dombrowski et al. 2008 | Use versus non-use of specific behaviour change techniques (BCTs) aimed at increasing *physical activity* | Stratified meta-analysis and uni-variate meta-regression of RCT data | N varies by BCT; 1) 2 to 20, typically 5 to 10 with BCT included (N=428 to 4592)  2) 1 to 10, typically 3 to 8 studies with BCT included (N=94 to 3595) | Weight (kg) | 1) active intervention: 1 to 14 mths, (mean 6 mths)  2) maintenance phase: 6 to 36 mths (mean 19mths) | 1) For active intervention, one BCT aimed at changing weight behaviour was significantly associated with net weight loss: T17 (*prompt practice*, -3.6 kg), explaining 34.3% of between study heterogeneity  2) For maintenance, two BCTs were significantly associated with net weight loss (T22 *prompt self talk*, -3.4 kg and T26 *time management*, -3.4 kg). However, both of these BCTs were only associated with one intervention. A positive non-significant trend was found for T12 (*prompt self-monitoring of behaviour*, -2.8 kg, p = 0.06) | 1) 2+  2) 2+ | 15 |
| Eakin et al. 2000 | Individual tailoring in interventions versus those without | Descriptive ‘vote-counting’ of significant results for groups of RCTs & quasi-experimental studies | 1) 10 (4170)  2) 7 (23,573) | Self-reported physical activity (effect size for continuous outcomes, Odds ratio for categorical outcomes) | 1) < 12 mths (median 6 wks)  2) >= 12 mths (median 12 mths) | 1) 6 of the 7 studies with significant short-term effects used a tailored intervention.  2) 1 of 3 studies with significant long-term effects was tailored. Overall, only 3 of 10 tailored studies had no significant effects | 2-  2- | 14 |
| Ogilvie et al. 2007 | Individual tailoring in interventions versus no tailoring | Descriptive summary of characteristics of successful interventions | nr (possibly 48) | Self-reported or pedometer-recorded walking (mins/week) | nr | Effective interventions typically involved content tailored to participants’ requirements or circumstances | Ungraded | 16 |
| Ogilvie et al. 2007 | Brief advice walking intervention, including goal-setting, versus no advice | Descriptive summary of individual RCT results | RCTs (1703) | Self-reported walking (mins/week) | 6 wks to 12 mths  (median 12 mths) | Intervention increased walking by (range) 0 to +44 min/wk (median 27min/wk), with a significant difference in 3 of 5 RCTs | 1+ | 16 |
| Bosch et al. 2007 | Contracts versus supervised exercise versus minimal contact | Descriptive summary of individual RCT results | 2 (159) | Weight (lbs) | 10 wks and 12 mths | One study showed no significant differences in weight loss between contracts and controls at 10 wks. The other found that people with contracts lost 3.5lbs more than those with minimal contact (p<0.05) at 12 weeks, but no significant difference at 12months (0.1 lbs) | Ungraded | 17 |
| Bravata et al. 2007 | Pedometer based intervention versus control | Meta-analysis 1) RCTs    2) Cohort studies | 1) 8 (277)    2) 18 (2490) | Walking (pedometer-recorded steps /day) | 1) 4 to 24 wks (median 10.5 wks)    2) 3 to 104 wks (median 10.5 wks) | Interventions which included pedometer use resulted in:-  1) Mean 2004 steps/day > controls (95%CI: 878 to 3129)    2) Mean 2183 steps/day > baseline (95%CI: 1571to 2796). NB: All but one small study (N=48) had follow-up of 36 weeks or less | 1) 1+  2) 2++ | 14 |
| Ogilvie et al. 2007 | Pedometer based intervention versus control | Descriptive summary of individual study results | 7 (652) (6 RCTs) | Walking (pedometer-recorded minutes/week) | 6 wks to 12 mths (median 13 weeks) | Interventions which included pedometer use resulted in (range = -11 to +181 min/wk; median 54min/wk) with a significant difference in 3 of 7 studies (2 of 6 RCTs). Studies not finding significance were considerably under-powered (N = 15 to 61) | 1+ | 16 |
| Richardson et al. 2008 | Pedometer based intervention | Descriptive summary of study effects in intervention arms only | 9 (307) inc. 4 RCTs | Walking (pedometer-recorded steps /day) | 4 wks to 1yr (median 16 wks) | Average daily step-count increased in all studies (Range 1827 to 4556 steps/day).This equates to between 1 mile and just over 2 miles, or an additional 20 to 40mins of walking/day | 2+ | 15 |
| Richardson et al. 2008 | Pedometer based intervention | Meta-analysis of intervention arms for RCTs and cohort studies | 9 (307) inc. 4 RCTs | Weight (kg) | 4 wks to 1yr (median 16 wks) | In the intervention arms, the pooled mean change from baseline was -1.27 kg (95% CI: -1.85 to -0.70). This equates to 0.05 kg/wk or 2.5 kg /year | 2+ | 15 |
| Bravata et al. 2007 | Pedometer based intervention | Meta-analysis of intervention arms only for RCTs and cohort studies | 18 (562) | BMI (kg/m^2^) | 3 to 104 wks (median 10.5wks) | In the intervention arms, BMI decreased by 0.38 (95% CI: 0.05-0.72; P=.03) from baseline to follow up | 2++ | 14 |
| Bravata et al. 2007 | Use of pedometer with or without step diaries | Stratified meta-analysis of data from intervention arms and cohort studies | 26 (2645) of which  3 (~950) had no step diary | Walking (pedometer-recorded steps /day) | 3 to 104 wks  (median 10.5 wks) | Interventions with pedometers not using a step diary did not significantly increase activity over baseline (mean 832 steps/day, 95% CI: −258 to 1922). Those using a diary significantly increased their activity over baseline (mean 2649 steps/day, 95% CI: 2032 to 3266) | 2- | 14 |
| Bravata et al. 2007 | Use of pedometers with or without step goals | Stratified meta-analysis of data from intervention arms and cohort studies | 26 (2645);  3 (77) with no step goal | Walking (pedometer-recorded steps /day) | 3 to 104 wks  (median 10.5 wks) | Interventions with pedometers not using a step goal did not significantly increase activity over baseline (686 steps/day, 95%CI: -1621 to 2994).  With the use of the 10 000-step-per-day goal steps-per-day increased significantly by more than 2988 (95%CI: 1646 to 4350) or 2363 (95%CI: 189 to 2936) for other step goals | 2- | 14 |
| Bravata et al. 2007 | Use of pedometers with or without step goals | Multi-variate meta-regression of data from intervention arms and cohort studies | 1) 26 (2645)  2) 18 (562) | 1) Walking (pedometer-recorded steps /day)  2) BMI (kg/m^2^) | 1) 3 to 104 wks  (median 10.5 wks)  2) 3 to 104 wks  (median 10.5 wks) | 1) Having a step goal was the main predictor of increased physical activity (p=.001). No other covariates (inc. gender, BMI, ethnicity, baseline activity) were significant  2) BMI change was significantly associated with having a step goal (p= 0.04), independently of other covariates (inc. gender, BMI, ethnicity, baseline activity) | 2+  2+ | 14 |
| Bravata et al. 2007 | Use of pedometers with or without physical activity counseling | Multi-variate meta-regression of data from RCT intervention arms and cohort studies | 26 (2645) | Walking (pedometer-recorded steps /day) | 3 to 104 wks  (median 10.5wks) | “Physical activity counseling” was not a significant predictor of increased physical activity. NB: This is poorly defined, with “some providing several weekly sessions to motivate walking and give individualised feedback, whereas others provided only a brief general physical activity lecture” | 2- | 14 |
| **3. Motivational interviewing** | | | | | | | | |
| Rubak et al. 2005 | Motivational interviewing based intervention vs. traditional advice-giving /usual GP care | Generic inverse variance meta-analysis of RCTs making this comparison | 6 (1140) | BMI | nr (but within range 3 to 24 mths) | The combined effect size estimate was 0.72 BMI units (95% CI: 0.33 to 1.11, p<0.0001). | 1++ | 16 |
| Burke et al. 2003 | Motivational interviewing based intervention versus control | Meta-analysis of RCTs making this comparison | 1) 4 (832)  2) 1 (523) | Standardised mean difference (Cohen’s d) for dietary and physical activity outcomes (mix of objective and self-reported) | 1) 12 to 18 wks  2) 12 mths | 1) The interventions produced moderate-to-strong effects (SMD= 0.53, 95%CI: 0.32 to 0.74), compared with standard treatment or placebo controls  2) In this single study, the increase in physical activity was not significant (SMD = 0.17, 95%CI: -0.12 to 0.46) | 1) 1+  2) 1- | 14 |
| **4. Targeting multiple behaviours** | | | | | | | | |
| Shaw et al. 2006 | Adding exercise to diet | Meta-analysis of RCTs making this comparison | 1) 15 (1079)  2) 6 (482) | 1) Weight (kg)  2) BMI (kg/m^2^) | 1) 3 to 12mths (median 16 wks)  2) 3 to 12 mths (median 6 mths) | 1) Adding exercise to diet produced additional weight loss of -0.65 kg (95%CI: -0.97 to -0.33)  2) Adding exercise to diet produced additional change in BMI of -0.31 kg/m^2^ (95%CI: -0.55 to -0.07) | 1) 1++  2) 1+ | 16 |
| Curioni & Lourenco 2005 | Adding exercise to diet | Meta-analysis of RCTs making this comparison | 1) 6 (407)  2) 6 (407) | Weight (kg) | 1) after intervention  2) 1yr after the end of the intervention | 1) Weight loss (±SD) for diet & exercise was approx 30% greater than for diet: -13.0 ±10.4 kg vs -9.9 ±9.6 kg, SMD = -0.20 (95%CI: -0.41 to 0.01, p=0.06)  2) Sustained weight loss (±SD) for diet & exercise was 50% greater: -6.7±8.3 kg vs. -4.5±11.3 kg SMD = -0.20 (95%CI: -0.42 to 0.01, p = 0.06) | 1-  1- | 14 |
| Avenell et al. 2004 | Adding exercise to diet | Meta-analysis of RCTs making this comparison | 1) 2 (269)  2) 2 (131)  3) 1 (21) | Weight (kg) | 1) 12mths  2) 18mths  3) 36mths | Adding exercise to diet produced additional weight loss (95%CI) of:-  1) -1.95 kg (-3.22 to -0.68),  2) -7.63 kg (-10.33 to -4.92)  3) -8.22 kg (-15.27 to -1.16) | 1) 1+  2) 1-  3) Ungraded | 16 |
| Dansinger et al. 2007 | Adding exercise to diet | 1) Multi-variate meta-regression  2) Description of individual RCTs with this comparison  3) T-tests of intervention arm means at different times  4) T-tests of BMI slopes in intervention arms at different times | 1) 46+ (6386)  2) 7 (1016)  3) N varies by time point (nr)  4) N varies by time point (nr) | Weight (kg) | 6-60 mths (median varies by analysis /nr) | 1) Adding exercise to dietary intervention made no significant difference at the end of the active or maintenance phases (p= 0.50, p=0.62) 2) Weight loss was generally greater among participants in combined diet & exercise programs than for diet alone. However, most differences were not significant 3) Active phase: At 12 mths, diet and exercise produced significantly greater weight loss than diet alone, but weight changes were similar in both groups at 3 and 6 mths. Maintenance phase: Changes in weight were not significantly different across studies 4) At 3-12 mths, the 3 diet & exercise interventions led to significantly greater weight loss than diet alone (Mean Diff: -0.23 kg/m^2^/mth, p=0.009). For other time periods slopes did not differ significantly | 1) 2-    2) 1-    3) 2-  4) 2- | 17 |
| Avenell et al. 2004 | Adding exercise to diet & behaviour therapy | Meta-analysis of RCTs making this comparison | 1) 7 (166)  2) 3 (237) | Weight (kg) | 1) 12mths  2) 24mths | Adding exercise to diet & behaviour therapy produced additional weight loss of:-  1) -3.02 kg (95%CI: -4.94 to -1.11)  2) -2.16 kg (95%CI: -4.20 to -0.12) | 1+1+ | 16 |
| Shaw et al. 2006 | Exercise versus diet | Meta-analysis of RCTs making this comparison | 7 (467) | Weight (kg) | 3 to12mths (median 26 wks) | Exercise produced less weight loss than dietary intervention by 3.6 kg (95%CI: 2.95 to 4.26) | 1- | 16 |
| Shaw et al. 2006 | High versus low intensity exercise with no dietary change | Meta-analysis of RCTs making this comparison | 4 (317) | Weight (kg) | 3.5 - 12mths (median 34 wks) | All trials favoured high intensity exercise. Weight loss was -1.47 kg (95%CI: -2.28 to -0.66) greater in the high intensity exercise group | 1- | 16 |
| Shaw et al. 2006 | High versus low intensity exercise with dietary change | Meta-analysis of RCTs making this comparison | 7 (224) | Weight (kg) | 12 to 20wks (median 16 wks) | Weight loss was not significantly greater in the high intensity exercise group -0.08 kg (95%CI: -1.20 to 1.04). | 1- | 16 |

Abbreviations: BCT = Behaviour Change Technique, CBT = Cognitive behavioural therapy,. RCT = Randomised Controlled Trial. SMD = Standardised Mean Difference. BMI = Body Mass Index (Kg/m2). SD = Standard Deviation. OQAQ = Oxman Quality Assessment Questionnaire, Mean Diff = Mean difference, NR = not reported, Mths = Months, Wks = Weeks, GP = General practitioner, NB = nota bene.

| **Table S10: Mode of Delivery** | | | | | | | | |
| --- | --- | --- | --- | --- | --- | --- | --- | --- |
| **Study** | **Comparisons** | **Method of comparison** | **N studies (N participants)** | **Outcome** | **Follow-up time** | **Results** | **Evidence Grade** | **OQAQ Review Quality**  **(out of 18)** |
| Avenell et al. 2004 | Group versus individual (one-to-one) intervention | Meta-analysis of RCTs making this comparison | 1) 4 (94)  2) 1 (17)  3) 1 (58)  4) 1 (53) | Weight (kg) | 1) 12mths  2) 18mths  3) 24mths  4) 60mths | Net weight loss in favour of group intervention mode:  1) 1.59 kg (95% CI: -1.81 to 5.00)  2) -0.74 kg (95% CI: -4.21 to 5.69)  3) 8.10 kg (95% CI: 2.19 to 14.01)  4) 4.40 kg (95% CI: -3.51 to 12.31) | 1, 2, 3 & 4) Ungraded | 16 |
| Michie et al. 2008 | Group versus individual (one-to-one) or mixed mode intervention | Multi-variate and uni-variate meta-regression of RCT data | 71 (28,838) | Objective or self-report of behavior change (diet and physical activity) | 1 wk to 24 mths (mean 6 mths) | Delivery mode was not significantly associated with behaviour change (p > 0.05) | 2+ | 15 |
| Dombrowski et al. 2008 | Group versus individual (one-to-one) or mixed mode intervention | Stratified meta-analysis of RCT data and comparison of groups using meta-regression | 11 (1108)  6 (822)  6 (3090) | Weight (kg) | Active intervention phase (nr. Estimate mean 6 mths) | One-to-one interventions were less effective than group (p=0.07) or combined modes (p=0.05). Groups delivered -4.0 kg (95% CI: -6.1 to -2.2), one-to-one -0.9 kg (95% CI: -1.5 to -0.4) and combined -3.8 kg (95% CI: -5.4 to -2.1) | 2+ | 15 |
| Dombrowski et al. 2008 | Group versus individual (one-to-one) or mixed mode intervention | Stratified meta-analysis of RCT data and comparison of groups using meta-regression | 2 (84)  2 (148)  7 (3457) | Weight (kg) | Maintenance phase (nr. Estimate mean 19mths) | One-to-one interventions were not significantly different from group or combined modes. Groups delivered -5.1 kg (95% CI: -9.5 to -0.8), one-to-one -4.0 kg (95% CI: -5.8 to -2.2) and combined -3.1 kg (95% CI: -5.3 to -1.0) | 2+ | 15 |
| McTigue et al. 2003 | Group versus individual (one-to-one) intervention | Descriptive review of study characteristics | nr | Weight (kg) | 12 to 34 mths (median 12 mths) | Treating patients on an individual rather than a group basis seemed less important than intervention intensity | 2- | 16 |
| Ogilvie et al. 2007 | Group versus individual (one-to-one) intervention | Descriptive review of study characteristics | 27 (8764) | Self-reported or pedometer-recorded walking (minutes/week) | 6 wks to 10 yrs (median 6mths) | Both individual and group approaches seem capable of delivering modest changes in physical activity | 2- | 16 |

Abbreviations: RCT = Randomised Controlled Trial. OQAQ = Oxman Quality Assessment Questionnaire, NR = not reported, Mths = months, Wks = Weeks.

| **Table S11: Intervention Provider** | | | | | | | | |
| --- | --- | --- | --- | --- | --- | --- | --- | --- |
| **Study** | **Comparisons** | **Method of comparison** | **N studies (N participants)** | **Outcome** | **Follow-up time** | **Results** | **Evidence Grade** | **OQAQ Review Quality**  **(out of 18)** |
| Michie et al. 2008 | Medically or non-medically trained health professional, or non-health professional | Multi-variate & uni-variate meta-regression of RCT data | 71 (28,838) | Objective or self-report of behaviour change (diet and physical activity) | 1 wk to 24 mths (mean 6 mths) | Intervention provider had no statistically significant association with behaviour change (p > 0.05) | 2+ | 15 |
| Dombrowski et al. 2008 | Professional, layperson, or both | Stratified meta-analysis of RCT data and comparison of groups using meta-regression | 11 (1263)  5 (509)  7 (3248) | Weight (kg) | Active intervention phase: 1 to 14 mths (mean 6.2) | Professionals -2.7 kg (95% CI: -4.2 to -1.2); lay people -2.9 kg (95% CI: -4.9 to -1.0); lay people with professionals -4.1 kg (95% CI: -7.2 to -1.1). No significant difference between intervention providers (p > 0.05) | 2+ | 15 |
| Eakin et al. 2000 | Physicians, nurses, health educators or public health students | Descriptive review of study characteristics | 15 (26,219) | Self-reported physical activity | 1) < 12 mths (median 6 wks)  2)>=12 mths (median 12 mths) | No clear association was found between type of intervention provider and effectiveness | 2- | 14 |
| Ogilvie et al. 2007 | Doctor, nurse, exercise specialist, or other | Descriptive review of study characteristics | 27 (8764) | Self-reported or pedometer-recorded walking (mins/week) | 6 wks to 10 yrs (median 6mths) | No clear relationship was found between type of intervention provider and effectiveness | 2- | 16 |
| Thompson et al. 2003 | Dietician versus self-help resources | Meta-analysis of RCTs making this comparison | 4 (588) | Weight (kg) | 26 wks to 2yrs (median 12 mths) | There was no significant difference between dietician and self-help (-0.42 kg, 95% CI: -1.0, 0.2). This may reflect poor study design and other confounding factors noted by the authors | Ungraded | 18 |
| Thompson et al. 2003 | Dietician versus counsellor | Meta-analysis of RCTs making this comparison | 1 (78) | Weight (kg) | 12 mths | One small study showed a significant difference in favour of dieticians (-5.8 kg, 95% CI: -8.91, -2.69) | Ungraded | 18 |

Abbreviations: RCT = Randomised Controlled Trial. OQAQ = Oxman Quality Assessment Questionnaire, Mths = Months, Wks = Weeks

| **Table S12: Intervention Intensity** | | | | | | | | |
| --- | --- | --- | --- | --- | --- | --- | --- | --- |
| **Study** | **Comparisons** | **Method of comparison** | **N studies (N participants)** | **Outcome** | **Follow-up time** | **Results** | **Evidence Grade** | **OQAQ Review Quality**  **(out of 18)** |
| **1. Changes in weight or BMI** | | | | | | | | |
| Shaw et al. 2005 | More intensive versus less intensive behavioural intervention (based on frequency of contacts, duration of intervention or no. of behavioural strategies) | 1) Meta-analysis of RCTs making this comparison  2) Single RCT comparing different intensities | 1) 10 (306)  2) 1 (58) | Weight (kg) | 1) <= 12mths (median 7 mths)  2) 30 mths | 1) Eight studies favoured more intensive behavior therapy and two studies favoured less intensive behaviour therapy. More intensive intervention produced 2.3 kg more weight loss (95% CI: 1.4 to 3.3)  2) Intensive intervention -1.6 kg, less intensive intervention -1.4 kg (p = 0.45) | 1) 1+  2) Ungraded | 17 |
| Shaw et al. 2005 | More intensive versus less intensive behavioural intervention (based on frequency of contacts, duration of intervention or no. of behavioural strategies) | Descriptive summary of RCTs with different intervention intensities (not suitable for meta-analysis) | 6 (390) | Weight (kg) | 1) <= 12mths (median 7 mths) | In 4 studies high intensity intervention produced greater weight loss, in 2 studies low intensity produced greater weight loss. Weight loss ranged from 1.4 to 8.4 kg in high intensity and 0.9 to 10.5 kg in low intensity interventions | 2+ | 17 |
| McTigue et al. 2003 | Number of contacts in first 3 months in relation to weight change in RCTs and other studies | Descriptive summary of groups of studies with different intensities | 11 (7425) | Weight (kg) | 12 to 54 mths  (median 12 mths) | Higher intensity was associated with increased effectiveness, but this is almost perfectly confounded with use of BCTs (see Table 4). The mean weight loss for higher intensity (and behavioural) interventions ranged from 3 to 5 kg more than controls. In studies with true control groups, the mean weight loss was (range) 2.5 to 5.5 kg for high intensity (and behavioural) interventions and 0.2 to 0.9 kg for low and medium intensity (and non-behavioural) interventions | 2- | 16 |
| Norris et al. 2007 | Number of contacts in relation to net weight change | Multi-variate meta-regression of RCT data | 9 (5137) | Weight (kg) | 1-10yr (mean 3.2yrs) | The total number of intervention contacts correlated significantly with a decrease in weight (p = 0.015) | 2+ | 17 |
| Tsai & Wadden 2005 | Number of sessions attended in relation to weight change | Descriptive summary of RCT findings | 1 (148) | Weight (%) | 2yrs | Participants who attended the most group sessions over 2 yrs maintained the largest weight loss | 2- | 15 |
| McTigue et al. 2006 | Frequency of monthly contacts during the first 3 months in relation to weight change in RCTs and other studies | Descriptive summary of groups of studies with different intensities | 10 (nr) | Weight (kg) | 12 to 48 mths  (median 15 mths) | In 7 controlled trials of higher intensity intervention, 3 found significant weight loss of 3-4 kg more than control at 18-30 mths, 1 found borderline significance (-2 kg) and 3 found no significant difference (although 2 were comparisons between interventions rather than vs. controls). Of 3 studies with lower intensity intervention, only 1 showed significant weight loss (-1.4 kg) | 2- | 16 |
| Dombrowski et al. 2008 | Contact frequency  1) above median  2) median  3) below median | Stratified meta-analysis of RCT data and between group comparison using uni-variate meta-regression | 1) 7 (847)  2) 7 (484)  3) 9 (3689) | Weight (kg) | Active intervention phase: 1 to 14 mths (mean: 6.2mths) | Above median -3.6 kg (95% CI: -6.1 to -1.2), median -3.7 kg (95% CI: -6.4 to -1.0), below median -2.3 kg (95% CI: -4.2 to -0.3). No significant differences for median or above median compared with low frequency (p > 0.70) | 2+ | 15 |
| Dansinger et al. 2007 | Frequency of meetings during intervention in relation to weight change in  1) Active phase  2) Maintenance phase | Multi-variate meta-regression of RCT data | nr (46 (11,853) with about 15% insufficient data) | BMI (kg/m^2^) | Active phase: <= 12 mths  Maintenance phase: 12 to 60 mths | 1) During the active phase more scheduled support meetings were independent predictors of greater weight loss (p= 0.009)  2) During the maintenance phase, the frequency of meetings in the first year was not a predictor of weight change (p= 0.29) | 1) 2++  2) 2++ | 17 |
| Richardson et al. 2008 | Duration of intervention in relation to weight change in pedometer-based walking interventions | Multi-variate meta- regression of RCT data | 9 (307) | Weight (kg) | 4 wks to 1yr (median 16 wks) | Duration of intervention was significantly associated with increased weight change (β = -0.05; p = .003) | 2+ | 15 |
| Norris et al. 2007 | Duration of intervention in relation to net weight change in diet and /or physical activity interventions | Multi-variate meta-regression of RCT data | 9 (5137) | Weight (kg) | 1 to 10yrs (mean 3.2yrs) | There was no significant association between intervention duration and weight change. However, any association with duration may have been captured by the co-variate ‘total no. of contacts’ (see below) | 2+ | 17 |
| Bravata et al. 2007 | Duration of intervention in relation to weight change in RCTs and cohort studies of pedometer-based walking interventions | Multi-variate meta-regression of intervention arms | 26 (2645) | BMI (kg/m^2^) | 3 to 104 wks  (median 10.5 wks) | BMI change was associated with increased intervention duration (p=0.07 trend only) | 2- | 14 |
| **2. Changes in diet** | | | | | | | | |
| Brunner et al. 2007 | Number of personal contacts in relation to weight change | Stratified meta-analysis of groups of RCTs with different intensities | 1) 20 (6170)  2) 18 (8416) | Self-reported dietary change  1) Fat  2) Fruit & vegetable intake | 3mths to 4 yrs  (median 12mths) | 1) High intensity interventions produced significantly higher reductions in total dietary fat (-5.72% (95% CI: -7.75 to -3.69) vs. -1.68% (95% CI: -3.13 to -0.23) with high heterogeneity in the high intensity subgroup  2) A similar pattern was seen for reported fruit and vegetable intake (data not reported) | 1) 2-  2) 2- | 15 |
| **3. Changes in physical activity** | | | | | | | | |
| Bravata et al. 2007 | Duration of intervention in relation to weight change in RCTs and cohort studies of pedometer-based walking interventions | Multi-variate meta-regression of intervention arms | 26 (2645) | Physical activity | 3 to 104 wks  (median 10.5 wks) | Intervention duration was not a significant predictor of physical activity | 2- | 14 |
| Eakin et al. 2000 | Brief (3 to 10 minutes physical activity counselling) versus more lengthy intervention (15 to 120 minutes of multiple risk factor counselling) | Descriptive summary of groups of RCTs and other studies with different intensities | 10 (4170) | Self-reported physical activity levels | Up to 12 mths (median 6 wks) | 5 out of the 7 studies with significant short-term effects involved brief counselling sessions | 2- | 14 |
| **4. Combined outcomes** | | | | | | | | |
| Burke et al. 2003 | High dose intervention (>60 mins) versus low dose (5 to 60 mins) | Stratified meta-analysis of RCT data | 4 (366) with 1(84) low dose | Standardised mean difference in combined physical activity & dietary intake (Cohen’s d) | 12 to 18 wks (median 15 wks) | High dose interventions seemed to deliver stronger effects (SMD=0.69, no CIs reported) than lower intensity of intervention (SMD=0.03 no CIs reported) | 2- | 14 |

Abbreviations: RCT = Randomised Controlled Trial. OQAQ = Oxman Quality Assessment Questionnaire, SMD = Standardised mean difference, BCT = Behaviour change technique, NR = Not reported, Mths = Months, Wks = Weeks.

| **Table S13: Population Characteristics** | | | | | | | | |
| --- | --- | --- | --- | --- | --- | --- | --- | --- |
| **Study** | **Comparisons** | **Method of comparison** | **N studies (N participants)** | **Outcome** | **Follow-up time** | **Results** | **Evidence Grade** | **OQAQ Review Quality**  **(out of 18)** |
| Shaw et al. 2006 | Gender:  male versus female | Stratified meta-analysis of single-gender trials | 9 (100 males, 367 females) | Weight (kg) | 12 to 24 wks (median 12 wks) | No apparent difference in effectiveness (in trials of diet and exercise versus diet) between trials with male (-0.23 kg, 95% CI: -0.68 to 0.23) and female (-0.55, 95% CI: -1.26 to 0.16) participants | 2+ | 16 |
| Dombrowski et al. 2008 | Gender:  male versus female | Stratified meta-analysis of women-only and mixed-gender trials | 6 (556 women)  17 (4464 mixed populations) | Weight (kg) | Active intervention phase: Range: 1 to 14 mths, mean: 6.2mths | Weight loss in women-only studies (-2.6 kg, 95% CI: -5.4 to -2.1) was similar to that for mixed-sex studies (-3.1 kg, 95% CI: -4.6 to -1.6) and not significantly different (p>0.05) | 2+ | 15 |
| Foster et al. 2005 | Gender:  male versus female | Descriptive summary of within-RCT sub-group analyses | 8 (3024) | Self-reported physical activity, cardio-respiratory fitness | min. 6 mths (median not available) | Greater improvements in cardio-respiratory fitness for women were found in 3 studies, while 2 reported greater benefits in men (1 for cardio-respiratory fitness and 1 for physical activity) and 3 found no difference | 2- | 17 |
| Whitlock et al. 2003 | Gender:  male versus female (dietary behaviour) | Descriptive summary of RCTs & other studies, examining gender effects (by sub-group or regression analysis) | 9 (7524) | Self-reported physical activity or dietary outcomes | 6 to 18 mths (median 12 mths) | No substantial differences were found between men and women in 8 of 9 studies. In one study (despite no differences in other outcomes) women showed “modestly larger” self-reported intake of both fruit and vegetables and fat | 2- | 14 |
| Whitlock et al. 2003 | Gender:  male versus female (physical activity) | Descriptive summary of RCTs & other studies, examining gender effects (by sub-group or regression analysis) | 5 (6315) | Cardio-respiratory fitness & self-reported energy expenditure | 6 to 24 mths (median 16 mths) | Only one study reported any gender effects in that women (but not men) receiving more intensive intervention had significantly greater effects than less intense intervention (on expended energy at 6 (but not 12 & 24 months) mths and on cardiorespiratory fitness at 24 mths) | 2- | 14 |
| Dansinger et al. 2007 | People with type 2 diabetes versus those without | Multi-variate meta-regression of RCT data | 46 (6386), 10 with participants with diabetes | BMI (kg/m^2^) | Active intervention 3 to 36 mths (median 12 mths)  Maintenance phase 6 to 60 mths (median 18 mths) | Not having diabetes was an independent predictor of weight loss (or slower weight regain) during the active intervention (p< 0.001) and maintenance phases (p< 0.012)    At 3 mths (-0.47 vs. -1.19 kg/m^2^), 6 mths (-0.75 vs. -1.56) and 12 mths (-1.19 vs -2.04) of active intervention, studies of participants with diabetes reported about half the net weight loss for studies of participants without diabetes (p<.0.001)  Findings were controlled for frequency of support meetings, recommended calorie intake, type of intervention (Diet vs Diet + Exercise), attrition and methodological quality | 2++ | 17 |
| Dombrowski et al. 2008 | People with 1) type 2 diabetes (t2d) 2) t2d-related conditions (eg. pre-diabetes) 3) CVD-related conditions 4) other conditions (eg. cancer) | Stratified meta-analysis by group and between group comparison using meta-regression techniques. All based on RCT data | 44 (10,560) | Weight (kg) | End of active intervention (mean 6.2 mths) | Weight loss was lowest in people with t2d (-1.2 kg, 95% CI: -0.2 to -2.1) and highest in those with t2d-related conditions (-5.5 kg, 95% CI: -1.8 to -9.2). Weight loss for people with t2d was significantly lower than for t2d-related (p<0.005) and CVD-related co-morbidities (p=0.05) | 2+ | 15 |
| Galani & Schneider 2007 | Lifestyle intervention versus standard care in people with high cardiovascular risk | Meta-analysis of RCTs | 5 (1910) | Weight (kg) | Mean 36 mths | Obese and overweight people with CV risk factors achieved a net mean weight loss of -2.30 kg (95% CI: -3.67 to -0.92) | 1++ | 16 |
| Galani & Schneider 2007 | Lifestyle intervention versus standard care in people with impaired glucose tolerance (IGT) | Meta-analysis of RCTs | 8 (3150) | Weight (kg) | Not stated (min. 12 mths, likely mean 36 mths) | Obese and overweight people with IGT achieved a net mean weight loss of -2.93 kg (95% CI: -4.35 to -1.52) | 1++ | 16 |
| Galani & Schneider 2007 | Lifestyle intervention versus standard care in overweight and obese people | Stratified meta-analysis of RCTs 1) Overweight and 2) Obese populations | 1) 13 (3566)    2) 17 (8013) | Weight (kg) &  BMI (kg/m^2^) | Mean 36 mths | Weight loss was achieved in both groups, but was numerically higher (no statistical comparison) in obese populations  1) –2.19 kg (95%CI: -2.81 to -1.57). BMI -1.11 kg/m^2^ (95% CI: -1.56 to -0.66)  2) –3.49 kg (95%CI: -4.70 to -2.27). BMI -1.33 kg/m2 (95% CI: -1.93 to -0.72) | 2+  1) 1++  2) 1++ | 16 |
| Foster et al. 2005 | Population characteristics in relation to effectiveness  1) age (above or below 70/75 yrs)  2) self-reported physical activity  3) overweight  4) chronic health condition | Description of within-RCT sub-group analyses | 1) 2 (457)  2) 2 (457)  3) 2 (457)  4) 1 (284) | Cardio-respiratory fitness & self-reported physical activity | min. 6 mths (median not available) | 1) No age effects were found  2) No differences were found between high and low baseline physical activity groups  3) One study found a greater increase in physical activity for overweight participants (BMI > 27). One found no difference for any of 4 BMI groups  4) No significant difference for less than two vs. two or more self-reported health conditions | 1) 2-  2) 2-  3) 2-  4) 2- | 17 |
| Bravata et al. 2007 | Age, gender, ethnicity, sedentary population, initial weight in pedometer-based walking interventions | Multi-variate meta-regression of data from RCT intervention arms and cohort studies | 26 (2645) | Pedometer measured physical activity (steps /day) | 3 to 104 wks  (median 10.5 wks) | Gender, BMI, ethnicity and baseline activity were not significant predictors of increased physical activity, although trends were identified for age and sedentary baseline physical activity | 2- | 14 |
| Bravata et al. 2007 | Changes in BMI in relation to age, gender, ethnicity, sedentary population, initial weight | Multi-variate meta- regression of data from RCT intervention arms and cohort studies | 18 (2645) | BMI (kg/m^2^) | 3 to 104 wks  (median 10.5 wks) | Decreased BMI was associated with older age (p =.001), increasing percentage of white participants (p =.009), having a step goal (p =.04), and longer intervention duration (p =.07). It was not significantly associated with baseline steps per day, changes in steps per day, sex, dietary counseling, or baseline BMI | 2- | 14 |
| Michie et al. 2008 | Disadvantaged/ low income target population (yes, no); Sedentary / obese target population (yes, no); women only (yes, no) | Multi-variate & uni-variate meta-regression of RCT data | 71 (28,838) | Objective or self-report of behavior change (diet and physical activity) | 1 wk to 24 mths (mean 6 mths) | None of the target population variables were significantly associated with intervention effectiveness | 2+ | 15 |
| Gillies et al. 2007 | Baseline BMI in relation to effectiveness of diet /physical activity interventions | Multivariate meta-regression on RCT data | 10 (5885) | Hazard ratio for progressing to diabetes | 1.8 to 4.6 yrs (mean 3.4 yrs) | Each unit increase in mean BMI at baseline was associated with a decrease in hazard ratio of −7.3% (95% CI: −13.6 to −0.9). This was independent of age and follow-up time | 2++ | 17 |
| Ogilvie et al. 2007 | Sedentary versus non-sedentary targeted populations | Descriptive / ‘vote counting’ summary of RCTs and other studies | 27 (8764) | Self-reported or pedometer-recorded walking (mins/week) | 6 wks to 10 yrs (median 6mths) | Many of the successful interventions to promote walking were targeted at sedentary people. 10 of 14 studies found significant effects (71%) compared with 5 of the 13 (38%) non-sedentary target interventions | 2+ | 16 |
| Shaw et al. 2006 | age: <45 yrs versus >45 yrs | Stratified meta-analysis of RCTs with different age groups | 12 (433 <45yrs; 268 >45yrs) | Weight (kg) | 12 to 52 wks (median 16 wks) | People with a mean age of less than 45 lost -0.44 kg (95% CI: -0.86 to -0.02) more in the exercise and diet group than the diet-only group People with a mean age over 45 yrs lost -1.12 kg (95% CI: -1.75 to -0.50) more in the exercise and diet group than the diet-only group | 2+ | 16 |
| McTigue et al. 2006 | Summary of data for older people | Descriptive summary of RCTs and other studies | 11 (nr) | Weight (kg) | 12 to 48 mths  (median 15 mths) | Of 11 studies, 4 found significant weight loss (typically 3-4 kg more than controls in RCT studies at 18-30 mths), 1 demonstrated weight loss of borderline significance (2 kg more than controls), 6 found no significant weight loss (although 2 were comparisons between interventions rather than with controls) | 1+ | 16 |
| Knowler et al. 2002 | Age:  1) <45 yrs  2) 45-59 yrs  3) 60+ | Analysis of effectiveness within sub-groups in a single RCT | 1 (3234)  N=  1) 1000  2) 1586  3) 648) | Incidence of type 2 diabetes (cases /100 person yrs) | 1.8 to 4.6 yrs (mean 2.8 yrs) | Incidence of type 2 diabetes reduced more as age increased. Age-group figures (with 95% CIs) were:  1) 48% (27 to 63)  2) 59% (44 to 70)  3) 71% (51 to 83) | 2+  1) 1+  2) 1+  3) 1+ | - |
| Knowler et al. 2002 | Ethnicity:  1) White  2) African American  3) Hispanic  4) American Indian  5) Asian | Analysis of effectiveness within sub-groups in a single RCT | 1 (3234)  N=  1) 1768  2) 645  3) 508  4) 171  5) 142 | Incidence of type 2 diabetes (cases /100 person yrs) | 1.8 to 4.6 yrs (mean 2.8 yrs) | Incidence of type 2 diabetes (with 95% CIs) was reduced by  1) 51% (35 to 63)  2) 61% (37 to 76)  3) 66% (41 to 80)  4) 65% (7 to 87)  5) 71% (24 to 89) | 1+ | - |

Abbreviations: RCT = Randomised Controlled Trial. OQAQ = Oxman Quality Assessment Questionnaire. CV = Cardiovascular, CVD = Cardiovascular Disease. BMI = Body Mass Index. IGT = Impaired Glucose Tolerance. T2D = Type 2 Diabetes, NR = Not reported, N = Number, Mths = months, Wks = weeks, Mins = Minutes.

| **Table S14: Intervention setting** | | | | | | | | |
| --- | --- | --- | --- | --- | --- | --- | --- | --- |
| **Study** | **Comparisons** | **Method of comparison** | **N studies (N participants)** | **Outcome** | **Follow-up time** | **Results** | **Evidence Grade** | **OQAQ Review Quality**  **(out of 18)** |
| Michie et al. 2008 | Community, primary care, or workplace setting in relation to RCT effectiveness | Multi-variate & uni-variate meta-regression | 71 (28,838) | Standardised effect size for objective or self-report measures of diet and physical activity | 1 to 14 mths (mean: 6.2mths) | Intervention setting was not significantly associated with changes in diet and /or physical activity | 2+ | 15 |
| Ashworth et al. 2005 | Home-based versus center-based physical activity intervention | Descriptive summary of a single RCT | 1) 1 (151)  2) 1 (143) | Self-reported physical activity (% adherence to prescribed physical activity programme) | 1) 1yr    2) 2yrs | 1) Home-based participants adhered to their exercise program significantly better than centre-based participants (79% vs. 53%; mean diff 26.1%, 95%CI: 15.9 to 36.3)  2) The difference between the home-based and the centre-based programs was mostly maintained at 2yrs (68% vs 36%; mean diff 31.4%, 95%CI: 18.3 to 44.5) | 1) 1-  2) 1- | 17 |
| Brunner et al. 2007 | Healthcare versus work place or community based dietary interventions | Stratified meta-analysis of RCTs conducted in different settings | 1) 20 (6170)  2) 19 (8469) | 1) Self-reported dietary fat (%)  2) Self-reported fruit and vegetable intake (servings /day) | 1) 3 to 48 mths (median 12 mths)  2) 6 to 48 mths (median 12 mths) | Trials in a healthcare setting tended to show 1) numerically greater reductions in dietary fat (-5.22%, 95%CI: -7.80 to -2.64 vs. -3.15%, 95%CI: -4.73 to -1.56) and 2) numerically greater increases in fruit and vegetable consumption (1.88 servings/day, 95%CI: 1.07 to 2.70 vs. 0.83 servings/day, 95%CI: 0.20 to 1.47) than trials in workplace /community settings | 1) 2-  2) 2- | 15 |
| Kahn et al. 2002 | Social support based physical activity interventions in community settings | Descriptive summary of results from RCTs and other studies | 1) 4(nr)  2) 3(nr)  3) 3(nr)  4) 4 (nr) | 1) Self-reported physical activity (time spent)  2) Self-reported physical activity (frequency)  3) Aerobic capacity (VO2 max)  4) Adiposity (BMI, waist-to-hip ratio or % body fat) | nr | Median net increase of 44.2% (IQR: 19.9% to 45.6%) in time spent in physical activity. Net median increases of 19.6% (IQR: 14.6% to 57.6%) in frequency of exercise/physical activity. Median net increase in aerobic capacity of 4.7% (IQR: 3.3% to 6.1%). Median net change in adiposity of -7.3% (IQR: -8.1% to -6.8%) | 1) 2-  2) 2-  3) 2-  4) 2- | 15 |
| Ogilvie et al. 2007 | Remote support in walking interventions by internet or telephone | Descriptive summary of results from RCTs and other studies | 3 (264) | Self-reported or pedometer-recorded walking (minutes/week) | 3 to 6 mths (median 3 mths) | A significant difference in walking was found in all 3 studies (Range: +32 to +62 mins/wk) | 2+ | 16 |
| Tsai & Wadden 2005 | eDiets.com compared with use of a behavioural weight loss manual | Descriptive summary of results from a single RCT | 1 (46) | Weight (kg) | 1yr | People using the weight loss manual lost significantly more weight than those using eDiets.com (4.0% vs. 1.1%, p = 0.04) | Ungraded | 15 |

Abbreviations: RCT = Randomised Controlled Trial. OQAQ = Oxman Quality Assessment Questionnaire, Mean diff = Mean difference, IQR = Interquartile range, BMI = Body mass index, VO2max = maximum volume of oxygen, Mins = minutes, Wks = Weeks, VS = Versus, NR = Not reported
